# Adsorption Mechanism in Crystalline Micropores: Multimodal Fluctuations, Metastability and Phase Transformations in Nanoconfinement

**DOI:** 10.1021/acsnano.5c16606

**Published:** 2026-01-27

**Authors:** Małgorzata Stankiewicz, Anthony Dorhauer, Kornel Roztocki, Volodymyr Bon, Bartosz Mazur, Carlos Wexler, Stefan Kaskel, Lucyna Firlej, Bogdan Kuchta

**Affiliations:** † Faculty of Chemistry, 214839 Wroclaw University of Science and Technology, Wybrzeze Wyspianskiego 27, 50-370 Wroclaw, Poland; ‡ Department of Physics and Astronomy, 14716 University of Missouri, Columbia, Missouri 65211, United States; § Materials Science and Engineering Institute,University of Missouri, Columbia, Missouri 65211, United States; ∥ Faculty of Chemistry, 49562 Adam Mickiewicz University, Uniwersytetu Poznańskiego 8, 61-614 Poznań, Poland; ⊥ Department of Inorganic Chemistry, Faculty of Chemistry and Food Chemistry, Dresden University of Technology, Dresden, 10169, Germany; # Laboratory Charles Coulomb, UMR CNRS 5221, 27037 University of Montpellier, 34095 Montpellier, France; ∇ Laboratory MADIREL, Aix-Marseille University—CNRS, 13013 Marseille, France

**Keywords:** metastability, adsorption mechanism, nanopores, MOFs, water, methane, carbon dioxide

## Abstract

Understanding molecular adsorption in microporous materials is key to advancing gas separation, storage, and catalysis. Here, we study CO_2_ and CH_4_ adsorption in crystalline metal–organic frameworks (IRMOF-1, 8, 10, and 14), emphasizing the emergence of metastable states. Molecular simulations reveal that adsorption is governed by a fine balance between fluid–fluid and fluid–framework interactions, leading to transitions between low- and high-density pore-filling states. These metastable features are highly sensitive to pore geometry and thermodynamic conditions, especially near the adsorbate’s triple point. In contrast, water adsorption displays more complex behavior: strong hydrogen bonding induces stable clusters, multiple free energy minima, and exceptionally slow equilibration. These features often escape conventional simulations. Our results underscore the importance of metastability in accurately modeling and designing advanced nanoporous materials for practical applications.

## Graphical abstract

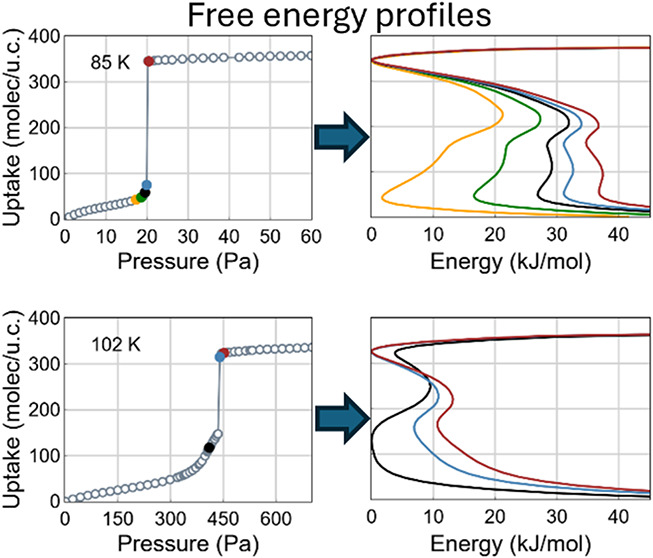

Adsorption of gases on the surfaces is often perceived as a conceptually simple phenomenon. Its fundamental principles were formulated by Irving Langmuir in 1918, who wrote: “*when gas molecules impinge against any solid or liquid surface, they do not in general rebound elastically, but condense on the surface, being held by the field of force of the surface atoms. These molecules may subsequently evaporate from the surface. The length of time that elapses between the condensation of a molecule and its subsequent evaporation depends on the intensity of the surface forces. Adsorption is the direct result of this time lag*”.^1^ This description intuitively captures the dynamic nature of the adsorbed phase, whose composition continuously fluctuates and is consistent with both thermodynamic and kinetic stability. While this definition remains valid across a wide range of environments, adsorption under nanoconfinement reveals additional, intriguing behaviors. One of them is the existence of metastability.

The analysis of sorption isotherms remains a fundamental and widely applied method for characterizing adsorption/desorption phenomena in a broad range of materials. This approach relies on the analysis of the isotherms’ shapes and their classification according to established schemes, such as those proposed by IUPAC.^2,3^ Although the IUPAC classification provides a valuable phenomenological framework, it offers only limited insight into the microscopic mechanisms driving sorption processes. These mechanisms are often complex and system-specific, emerging from the interplay of multiple factors, including structural heterogeneity of the adsorbent surface, a broad distribution of pore sizes and geometries, and spatial variations in the adsorption energy landscape. This complexity is particularly pronounced in crystalline microporous materials. This aspect has also been emphasized in the recent paper discussing adsorption hysteresis in nanoporous MOFs (Metal–Organic Frameworks)^4^ revealing two different mechanisms of rapid changes in the adsorption uptake: one resembling capillary condensation (gas-to-liquid transition) and the other more akin to liquid-to-solid transition.

According to IUPAC classification, microporous systems—defined by pore sizes below 2 nm—are associated with Type I adsorption isotherms, characterized by a steep slope at very low relative pressures, indicative of rapid pore filling. However, both experimentally measured and computationally derived adsorption isotherms frequently show significant deviations from this idealized Type I behavior in such systems.^4–10^ These deviations are particularly pronounced in materials with crystallographic symmetry and/or well-ordered pore-wall structures. They primarily result from heterogeneities in the adsorption energy landscape and from the complex interplay between different interaction components, modulated by the thermal energy of the surrounding environment. In particular, strong fluid–fluid interactions can compete with fluid-framework interactions, potentially giving rise to metastable states^8^ and specific pore filling mechanisms, including structural transitions.^9^

Metastability plays a critical role in molecular simulations. Systems that become trapped within local—rather than global-thermodynamic minima are described as kinetically stable or persistent (long-lived). Although such metastable states may be unobservable on macroscopic experimental time scales, which typically range from minutes to hours, they are often essential for interpreting microscopic behavior. In molecular simulations, which are generally limited to nanosecond time scales, metastability can significantly hinder reaching thermodynamic equilibrium, increase statistical uncertainty, and substantially increase the computational cost required to sample representative equilibrium ensembles.^11^

In this study, we investigate the emergence of metastable states during gas adsorption in microporous materials. We show that this phenomenon is common across ordered or crystalline microporous systems, including metal–organic frameworks (MOFs) and zeolites. The formation of metastable states is attributed to a confluence of factors: the spatial distribution and crystallographic symmetry of adsorption sites, the restricted pore dimensions (typically 1–2 nm), and the competitive interplay between gas–gas and gas–framework interactions. Together, these factors create a complex free energy landscape characterized by multiple local minima, leading to nontrivial adsorption pathways and mechanisms.^4,8^

The formation of metastable states during gas adsorption in microporous systems is analyzed using methane and carbon dioxide adsorption in selected model IRMOF structures. Our results demonstrate that the structural features and crystallographic symmetry influence the pore-filling process, frequently leading to metastable configurations in which the system can be temporarily trapped. The lifetimes of these states are found to be temperature-dependent, reflecting a delicate balance between thermodynamic driving forces and kinetic barriers. These findings underscore the intricate and dynamic nature of adsorption in nanoporous crystalline materials and provide new insights into the mechanisms governing molecular confinement and transport in such systems.

## Metastability: Fundamental Element of Multimodal Adsorption Mechanism

In *mesoporous* materials (pore dimensions >2 nm), a well-documented example of a metastability-driven phenomenon is the hysteresis observed becease of capillary condensation, consisting in rapid pore filling. In contrast, *microporous* materials exhibit different filling mechanisms.^8–18^ While capillary condensation is primarily governed by fluid–fluid interaction and often displays hysteresis, micropore filling is controlled by a more nuanced balance between fluid–fluid and fluid-framework interactions and is typically reversible. Moreover, micropore filling can induce structural transformations in the adsorbed phase,^4,9^ potentially leading to the formation of new stable phases or metastable intermediate states. This stands in contrast to capillary condensation in mesopores, where pores’ filling proceeds rapidly without altering the structure of the already adsorbed fluid layers.

Previous experimental and computational studies have reported atypical features in the adsorption behavior of methane and carbon dioxide in microporous MOF-5 (IRMOF-1) framework.^8,9,12,13^ Similarly, water adsorption isotherms in microporous materials have been shown to adopt a variety of shapes^12,14–19^ and are well-known to involve long living metastable states. In many crystalline microporous systems, adsorption isotherms deviate from the classical IUPAC Type I profile, characterized by rapid pore filling at very low pressures. Specifically, at low temperatures and pressures, the isotherms often exhibit an initial monolayer-like adsorption, followed by an S-shaped profile, and ultimately a sharp, step-like uptake.^13^ In our previous work,^9^ this abrupt pore filling was interpreted as the coexistence at nanoscale of low- and high-density adsorbate states, driven by correlated, large-amplitude fluctuations between these configurations. Such a mechanism requires the presence of at least one metastable state,^8^ with transitions between phases governed by a relatively low (and depending on temperature) free energy barrier separating the macroscopic low- and high-density phases (Figure 1). As a result, adsorption proceeds not through gradual, incremental accumulation of adsorbate molecules, but rather via a collective, synchronized, and cooperative structural transformation of the adsorbed phase. At higher temperatures, the free energy barrier between states of different density diminishes, leading to more frequent fluctuations and a gradual transition to a continuous adsorption profile.

**Figure 1:**
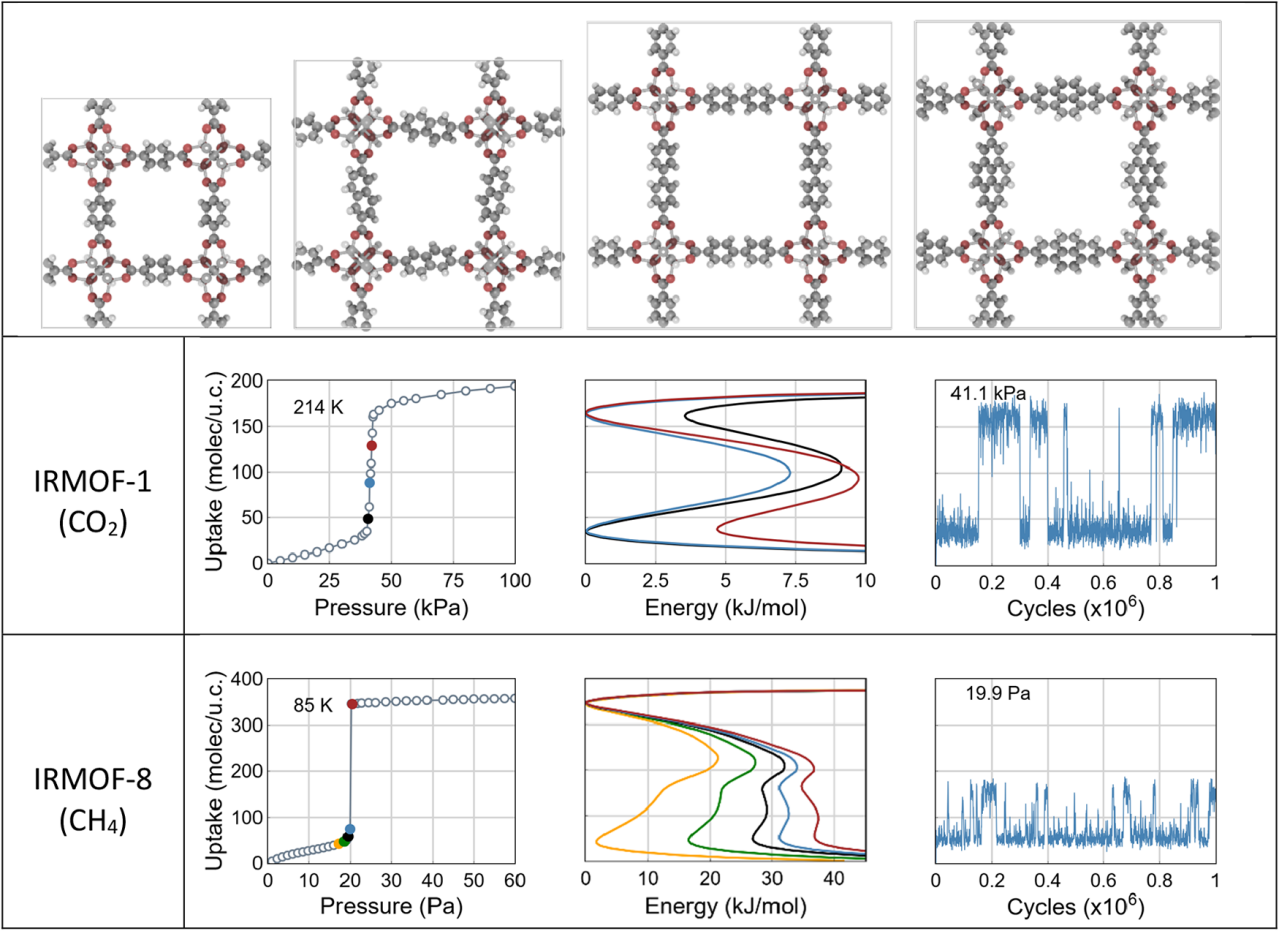
Structural representations of the cubic unit cells of IRMOF-1, 8, 10, and 14 (upper raw, left to right). All frameworks share the same Zn_4_O–carboxylate (pcu) topology, with increasing linker length leading to larger pores. The cubic unit cells show the axes length of *a* = 25.83, 30.09, 34.28, and 34.38 Å, respectively. Carbon atoms are shown in gray, oxygen in red, and hydrogen in white. Typical metastability (middle and bottom rows) observed for the CO_2_ and CH_4_ adsorption in IRMOFs. Numerical adsorption isotherms (left), free energy profiles (center), and uptake fluctuations (right) at pressures near pore filling. Middle row: two-state system (CO_2_ adsorption in IRMOF-1 at 214 K). Bottom row: three-state system (CH_4_ adsorption in IRMOF-8 at 85 K). These results illustrate the presence of metastable states and distinct adsorption pathways. A more detailed analysis of these behaviors is provided in the following sections. The energy given in kJ per mol of the adsorbent. These units are used consistently throughout manuscript unless stated differently.

Metastability plays a critical role in shaping the dynamics and kinetics of the adsorption process. In our previous paper,^8^ we demonstrated how metastability between low-density and high-density methane phase structures within IRMOF-1 pores governs the adsorption mechanism of adsorbate. Building on these results, the present study investigates the evolution of metastability as a function of pore size and temperature in a series of IRMOF-type frameworks (IRMOF-X, where *X* = 8, 10, 14), for both methane (CH_4_) and carbon dioxide (CO_2_).

## Results

### CO_2_ Adsorption in IRMOF-X Materials (*X* = 1, 8, 10, 14)

We begin our analysis of adsorption mechanisms by examining CO_2_ adsorption in IRMOF-type structures. Figure 2 presents the experimental isotherms of CO_2_ and CH_4_ adsorption in IRMOF-1. These results serve as a reference for understanding the distinct adsorption behaviors observed in this class of materials and guide the interpretation of corresponding simulation results.

**Figure 2:**
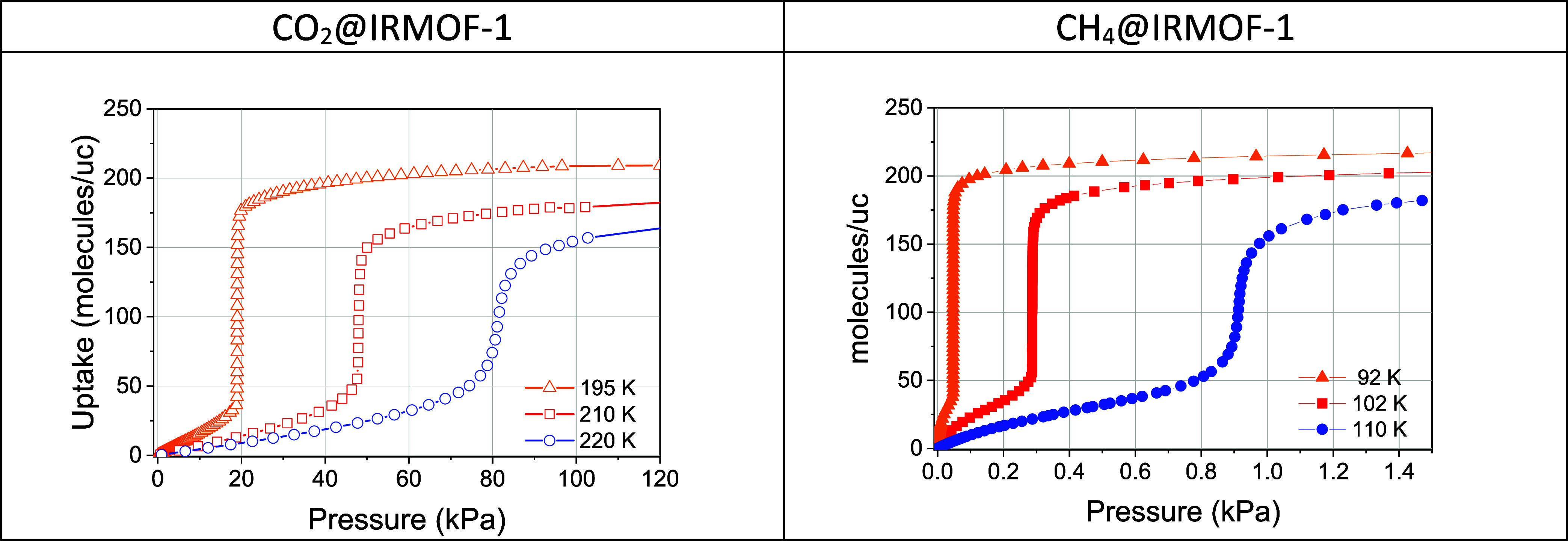
Temperature-dependent evolution of experimental adsorption isotherms for CO_2_ (left) and CH_4_ (right) in IRMOF-1. Adsorption and desorption points are indistinguishable. The CH_4_ data are reproduced from our previous work.^8^ The uptake units “molecules/uc” are naturally used for numerical results and they are used in this paper. To convert them into milliliters at STP per gram of MOF one needs to multiply by 3.63946 (We provide the experimental data in AIF format in Supporting Information).

At temperatures near the bulk triple point of CO_2_ (216.58 K, NIST database), the adsorption isotherms display a fully reversible, step-like character (Figure 2, left panel). A similar behavior is observed for CH_4_ adsorption at temperatures approaching its bulk triple point (90.67 K), as shown in Figure 2 (right panel).^8^ In both cases, increasing the temperature leads to a progressive transformation of the sharp, quasi-discrete transition into a smoother, S-shaped adsorption profile, indicating a more continuous adsorption process. The comparison of CO_2_ and CH_4_ adsorption isotherms suggests a shared underlying mechanism governing transitions between low- and high-density nanophases. This resemblance persists despite differences in molecular symmetry of guest molecules, adsorption conditions (temperature and pressure), and the absence of electrostatic interactions in CH_4_ adsorption. These findings point to *a degree of universality* in the adsorption mechanism that transcends specific structural and chemical properties of the adsorbate. This observation raises a fundamental question: what physicochemical parameters dictate the onset and nature of transitions between nanophases during pore filling in microporous materials?

TM-GCMC calculations of the free energy landscapes offer critical insight into this question.^8^ The free energy profiles (Figure 3, right column) were calculated as the logarithm of the number density distribution derived from TMMC simulations (see Methods section). In parallel, conventional Grand Canonical Monte Carlo method (GCMC) was used to compute adsorption isotherms and fluctuation properties. The calculated free energy reveals distinct minima over a specific pressure range, corresponding to low- and high-density adsorption states, separated by a finite energy barrier. When this barrier is low (here, below 10 kJ/mol for CO_2_), thermal fluctuations enable spontaneous transitions between the two states, at pressures corresponding to rapid pore filling (Figure 3, middle columns). Within the pressure range defining the step on the adsorption isotherm, the free energy consistently shows at least one metastable state. However, this state remains macroscopically undetectable, as the experimentally measured uptake represents a time-average value, weighted by the residence time in each of the coexisting states.^8^ As the temperature increases, the energy barrier progressively diminishes, and the two minima ultimately coalesce into a single, broader minimum. This marks the disappearance of distinct nanophases, and the onset of a continuous adsorption regime. We define the corresponding temperature as the *pore critical temperature T*_pc_.

**Figure 3:**
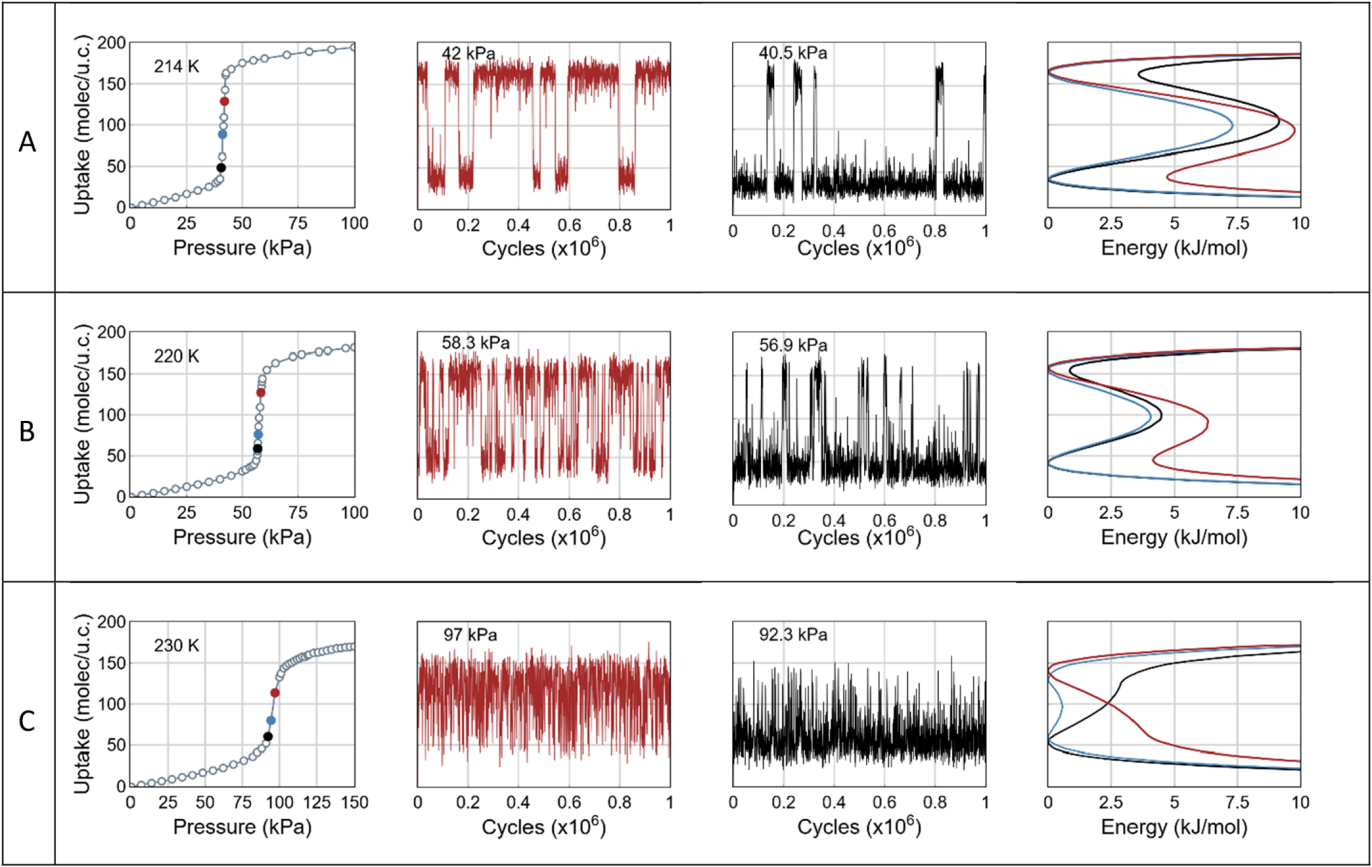
Adsorption isotherms (left), free energy (right), and bimodal fluctuations (center columns) of the CO_2_ uptake in IRMOF-1 at three temperatures: (A) *T* = 214 K, (B) 220 K, and (C) 230 K. Colored mark points on the isotherms indicate pressures at which the free energy profiles and uptake fluctuations in high density (red) and low-density (black) states are shown.

As an initial step toward generalization, we extended the analysis to CO_2_ adsorption in larger-pore IRMOF structures: IRMOF-8, IRMOF-10, and IRMOF-14. Structural parameters for all investigated frameworks are provided in Supporting Information (Table S1 in Supporting Information).

CO_2_ adsorption isotherms for all studied IRMOF-X materials at *T* = 220 and 230 K are presented in Figure 4. Qualitatively, the isotherms exhibit similar features across the series. However, the pressure range associated with the sharp, step-like adsorption in IRMOF-X (*X* > 1) is systematically shifted to higher values relative to IRMOF-1. This shift primarily reflects the variation of the accessible pore volume resulting from the incorporation of longer organic linkers. Interestingly, the shift between IRMOF-8 and IRMOF-14 isotherms is relatively small, despite the larger pore volume of IRMOF-14 (see Figure S2 in the Supporting Information). This behavior can be attributed to a compensating effect: the longer and more massive linker in IRMOF-14 introduces additional strong adsorption sites, which facilitate adsorption at lower pressures. For the same reason, the pressure shift between IRMOF-8 and IRMOF-10 is larger than that between IRMOF-8 and IRMOF-14, even though the unit cell volumes of IRMOF-10 and IRMOF-14 are comparable. In the case of IRMOF-10, the linker structure results in weaker adsorption forces, allowing the low-density phase to persist over a broader pressure range. Figures S3–S5 in the Supporting Information show the results of free energies and the corresponding bimodal fluctuations of the CO_2_ uptake in IRMOF-8, IRMOF-10 and IRMOF-14

**Figure 4:**
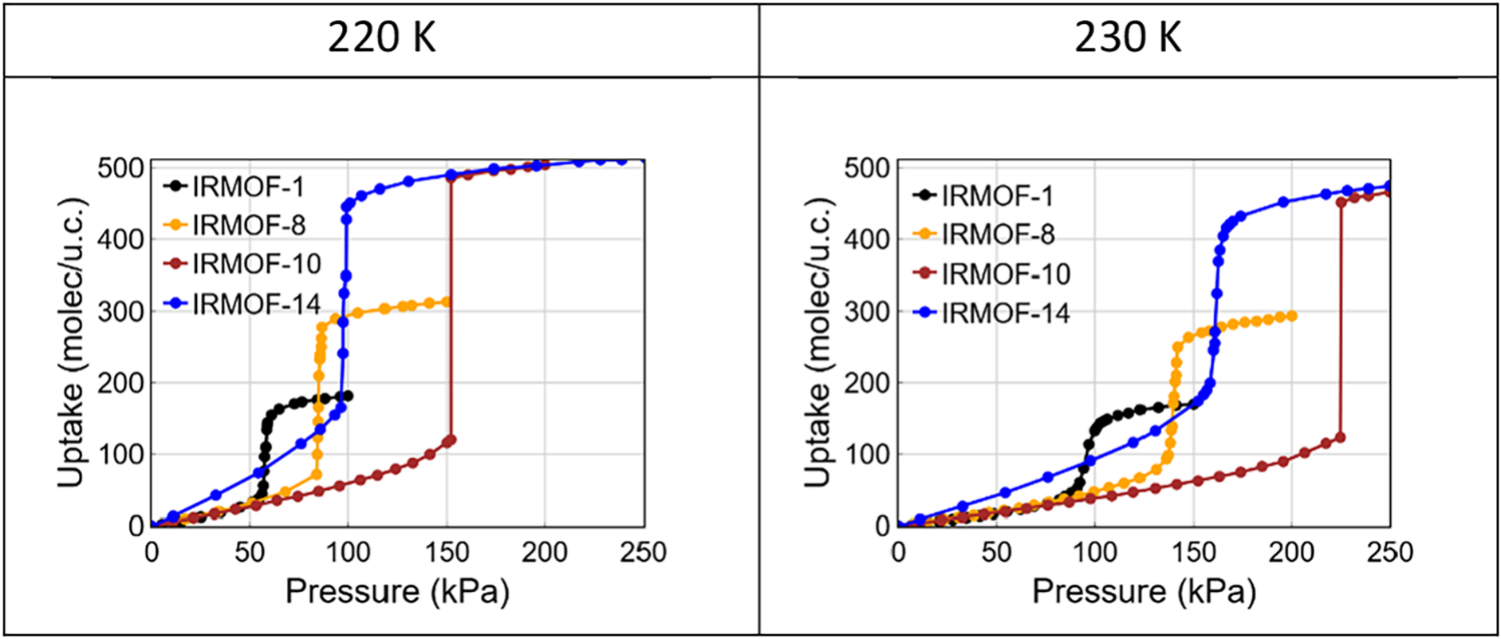
Simulated CO_2_ adsorption isotherms in IRMOF-1, IRMOF-8, IRMOF-10 and IRMOF-14 at *T* = 220 and 230 K. The shift of the pressure associated with the sharp step-like gas uptake reflects dependence on pore (accessible) volume and linker structure.

### Bimodal Behavior: The Limit of Reversible Pore Filling

The calculated free energy profiles (Figure 3) indicate that the bimodal behavior observed at pressures corresponding to step-like adsorption can be captured in Monte Carlo simulations when the energy barrier *E*_B_ separating the low- and high-density states remains sufficiently low (typically *E*_B_ < 10 kJ/mol for CO_2_). In this regime, the transition probability is high enough to allow fluctuations between the two states within the simulation time scale. To quantify this effect, we explicitly calculated the probabilities of crossing the barrier, assuming the transition probability between the low-density state (with energy *E*_L_) and the high-density state (with energy *E*_H_) as a two-step process. The total transition probability was expressed as the product of two subsequent transition probabilities, with the intermediate state defined by the barrier height *E*_B_

$$p\left(E_{L}\to E_{H}\right)=p\left(E_{L}\to E_{B}\right)p\left(E_{B}\to E_{H}\right)$$

$$p\left(E_{H}\to E_{L}\right)=p\left(E_{H}\to E_{B}\right)p\left(E_{B}\to E_{L}\right)$$

proportional to the Boltzmann factors when the final state has higher energy, and equal to 1 when the final state has lower energy

$$p\left(E_{L}\to E_{B}\right)=\exp\left(-\frac{{\Delta E}_{\mathrm{BL}}}{k_{B}T}\right)\leq 1$$

where Δ*E*_BL_ = *E*_B_–*E*_L_

$$p\left(E_{H}\to E_{B}\right)=\exp\left(-\frac{{\Delta E}_{\mathrm{BH}}}{k_{B}T}\right)\leq 1$$

where Δ*E*_BH_ = *E*_B_–*E*_H_

$$p\left(E_{B}\to E_{H}\right)=p\left(E_{B}\to E_{L}\right)=1$$

These transition probabilities satisfy the detailed balance (microscopic reversibility condition), which leads to the following relation

$$p\left(E_{L}\right)p\left(E_{L}\to E_{H}\right)=p\left(E_{H}\right)p\left(E_{H}\to E_{L}\right)$$

Therefore, we can estimate the transition probabilities *p*(*E*_L_ → *E*_H_) and *p*(*E*_H_ → *E*_L_)­

$$p\left(E_{L}\to E_{H}\right)=p\left(E_{L}\to E_{B}\right)p\left(E_{B}\to E_{H}\right)=p\left(E_{L}\to E_{B}\right)$$

$$p\left(E_{H}\to E_{L}\right)=p\left(E_{H}\to E_{B}\right)p\left(E_{B}\to E_{L}\right)=p\left(E_{H}\to E_{B}\right)$$

Figure 5 shows the estimated transition probabilities for IRMOF-X (*X* = 1, 8, 10, 14) at equilibrium pressures, i.e., when the free energies of the low- and high-density states are equal *E*_L_ = *E*_H_, plotted as a function of temperature. As the transition probability approaches *p* = 0.6, the distinction between the two well-defined nanophases disappears (see Figure S4, *T* = 260 K). The pore critical temperature, *T*_pc_, is defined as the temperature at which *p* = 1, corresponding to complete disappearance of free energy barrier.

**Figure 5:**
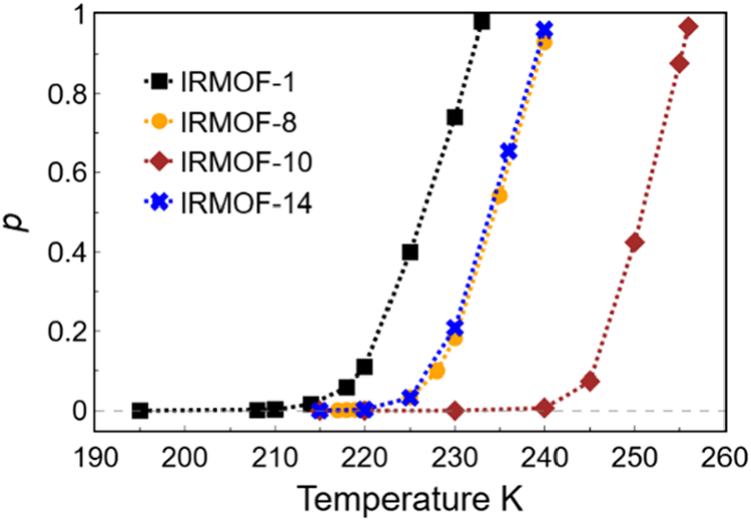
Estimated fluctuation probabilities (*p*) for CO_2_ adsorption in IRMOF-X (*X* = 1, 8, 10, 14) at equilibrium pressure (i.e., when the high- and low-uptake states have equal free energy, *E*_H_ = *E*_L_). As *p* approaches zero, observing fluctuations becomes increasingly rare and strongly dependent on the simulation length (typically requiring significantly more than ∼10^6^ Monte Carlo cycles).

The pore critical temperature *T*_pc_ defines the upper temperature limit at which bimodal adsorption behavior can be observed. This condition corresponds to the point where the free energy barrier separating low- and high-density states is comparable to, or smaller than, the thermal energy *k*_B_ *T*. Our results indicate that *T*_pc_ is governed primarily by two factors: pore size and linker topology. We observe an increase in the *T*_pc_ from IRMOF-1 to both IRMOF-8 and IRMOF-10, consistent with their larger pore volume. However, the *T*_pc_ of IRMOF-14 is approximately equal to that of IRMOF-8, despite IRMOF-14 requiring higher pressure for pore filling (Table 1). This suggests that, beyond pore size, the linker structure also plays a critical role in stabilizing specific adsorbed states.

**Table 1:** Triple Point (*T*_tr_) and Critical (*T*_c_) Temperatures for Bulk (CH_4_ and CO_2_), and Pore Critical (*T*_pc_) Temperatures for CO_2_ and CH_4_ Adsorbed in IRMOF-X (*X* = 1, 8, 10, 14)^a^

	*T*_tr_ (bulk)	*T*_pc_ (IRMOF-1)	*T*_*pc*_ (IRMOF-8)	*T*_pc_ (IRMOF-10)	*T*_pc_ (IRMOF-14)	*T*_c_ (bulk)
volume	---	17.23 nm^3^	27.25 nm^3^	40.29 nm^3^	40.64 nm^3^	---
CH_4_	90.67 K	∼110–115 K	∼125–130 K	∼120–125 K	∼125–130 K	190.56 K
CO_2_	216.58 K	∼230–235 K	∼235–240 K	∼255–260 K	∼235–240 K	304.13 K

a *T*_pc_ (IRMOF-X) is the lowest temperature where continuous transformation from the low density to high density adsorption state is observed (estimated accuracy of ± 4 K), that is, when the barrier between them disappears. Volume represents the mean crystallographic unit cell volume.

For CO_2_ adsorption, all studied IRMOFs exhibit qualitatively similar free energy profiles, each characterized by two states. In contrast, CH_4_ adsorption reveals more complex behavior in some frameworks, with free energies featuring multiple distinct minima indicative of additional metastable configurations.

### Metastability of CH_4_ Adsorption in IRMOF-X Materials (*X* = 1, 8, 14)

The mechanism of CH_4_ adsorption in IRMOF-1 has been recently investigated and reported in detail.^8^ Its free energy profile is qualitatively like that discussed above for CO_2_, exhibiting two well-defined metastable states. However, in larger pore frameworks, specifically IRMOF-8, IRMOF-10, and IRMOF-14, the adsorption behavior becomes more complex, with free energy landscape displaying multiple minima (Figure 1). This suggests that CH_4_ adsorption in these systems involves a series of structural transformations as a function of adsorption uptake. As the temperature increases, the adsorption landscape progressively smooths: the discrete minima begin to merge, and the energy barriers separating them decrease, ultimately leading to a more continuous adsorption process.

Figure 6 illustrates the key features of CH_4_ adsorption in IRMOF-8. At *T* = 85 K and 92 K, the free energy profile shows a multiminima structure within a very narrow pressure range, revealing the presence of intermediate configurations at the uptake of ∼150 molecules per unit cell (analogous to monolayer adsorption). These intermediate states are not thermodynamically stable and can only be inferred from the evolution of uptake fluctuations observed during the equilibration phase of simulations. Such free energy profiles may lead to nonphysical results in numerical simulations. For example, the fluctuations at *T* = 85 K resemble an equilibrated state, yet the high-density minimum in the free energy indicates that they correspond to a metastable configuration. By contrast, the fluctuations at *T* = 110 K (Figure 6, bottom row: *P* = 1365 Pa) represent a stable situation, despite appearing similar. The pressure range associated with these metastable states is extremely narrow and is not resolved in the corresponding adsorption isotherms. As temperature increases, the isotherms’ shape evolves. Metastable low-density states disappear, and uptake increases continuously with pressure.

**Figure 6:**
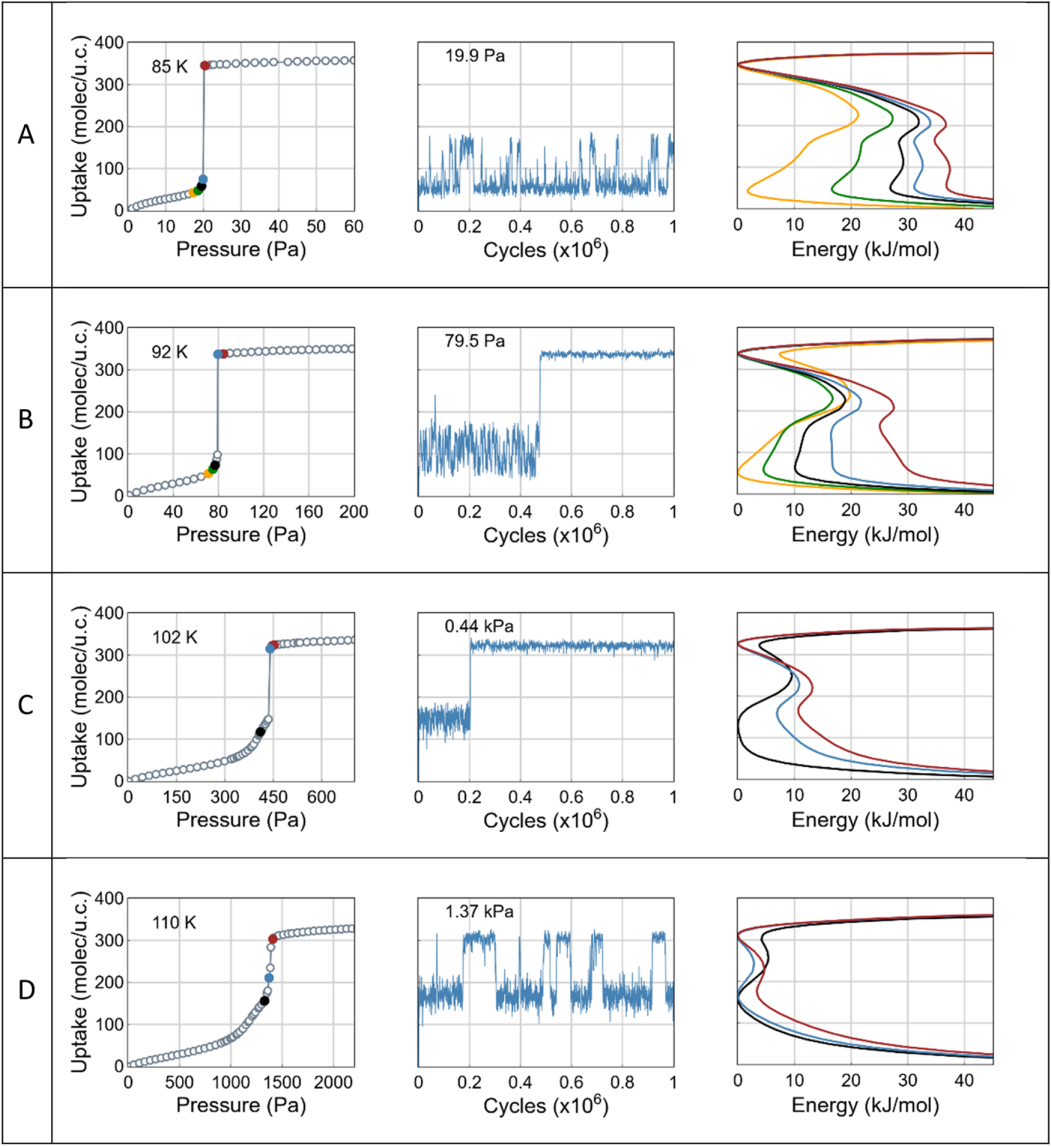
From left to right: adsorption isotherms, uptake fluctuations, and free energy profiles for CH_4_ adsorption in IRMOF-8, at *T* = 85 K, 92 K, 102 and 110 K. The uptake fluctuations (middle column) are shown at pressures: 19.9 Pa, 79.5 Pa, 440 and 1370 Pa, respectively for each temperature. Three colored uptake points on the isotherms and the blue color of the fluctuations correspond to the free energy curves of the same color. The fluctuations shown in rows (A–C) (*T* = 85, 92, 102 K, respectively) are taken from equilibration runs whereas the fluctuations at *T* = 110K (row D) represent the fully equilibrated uptake.

In IRMOF-14, CH_4_ uptake increases smoothly at low pressures until approximately half of the pore volume is filled (Figure 7). Within the temperature range of 115–125 K, two high-density adsorption states coexist in equilibrium, accompanied by large-amplitude uptake fluctuations in the upper part of the adsorption step. The adsorption mechanism resembles that of capillary condensation, where adsorption of initial layers is followed by more abrupt pore filling. However, the nature of the filling process varies with temperature. At lower temperatures (e.g., *T* = 115 K, Figure 7A, pore filling proceeds via more scattered events, while at higher temperatures (e.g., *T* = 125 K, Figure 7B), the process becomes more continuous.

**Figure 7:**
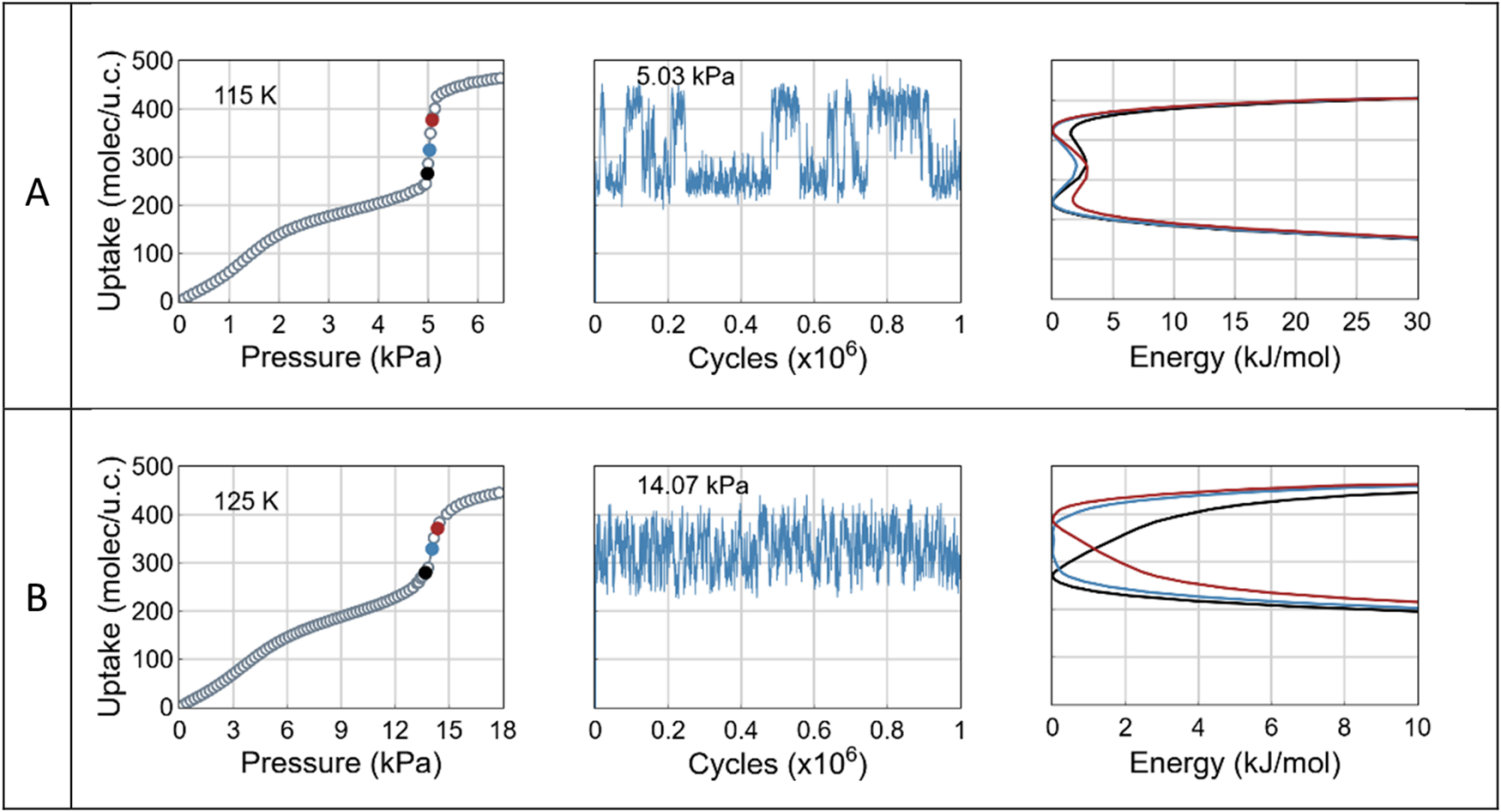
Adsorption isotherms, equilibrium uptake fluctuations and free energy profiles for CH_4_ adsorption in IRMOF-14 at (A) *T* = 115 and (B) 125 K. Colored points on isotherms indicate the pressures at which the free energy was calculated.

### Influence of the Size of Monte Carlo Simulation Box on Barriers between Metastable States

Two primary input parameters govern the outcome of the adsorption simulation. The first is the intermolecular potential model, which reflects the underlying physical interactions. The critical role of the model is well established and widely recognized (Figure S1).

The second parameter concerns the simulation methodology: the size of the simulation box. To evaluate the impact of box dimensions and geometry on the system’s tendency to become trapped in metastable, yet physically unrealistic states, we performed CH_4_ adsorption simulations in IRMOF-8 using Monte Carlo boxes of varying sizes. Three configurations were considered, corresponding to the following three-dimensional (*x*, *y*, *z*) unit cell arrangements: (1) 1 × 1 × 1, (2) 2 × 2 × 1, and (3) 3 × 3 × 1. These simulations were performed at a temperature of 110 K and a pressure of 1385 Pa.

The free energy profile for CH_4_ in the smallest simulation box (1 × 1 × 1) is shown in Figure 7 (right column), while those for larger boxes (2 × 2 × 1, and 3 × 3 × 1) are presented in Figure 8. In the smallest box, the equilibrium free energy landscape exhibits a barrier of approximately 3 kJ/mol and the system displays fluctuations between a medium density state (*N* ∼ 150) and a high-density state (*N* ∼ 300) during the simulation. As the box size increases, the free energy barrier also rises, reaching approximately 15 kJ/mol for the biggest box. According to the relation *p* ≈ exp­(−Δ*E*_B_/*k*_B_ *T*), the probability of observing fluctuations over such a barrier at *T* = 110 K is of the order of *p* ≈ 10^–8^. This vanishingly small probability effectively precludes the observation of fluctuations within standard MC simulations comprising ∼10^6^ steps. For comparison, in molecular dynamics simulations where the simulation time step is usually of the order of 0.5 fs, 10^6^ steps correspond to simulated times shorter than 1 ns. This limitation is purely technical and could be mitigated in longer simulations or with more powerful computer resources. In contrast, real experiments are not constrained by such restrictions and can probe systems over macroscopic time scales (minutes to hours), which are many orders of magnitude longer than those accessible in molecular simulations (nanoseconds to microseconds).

**Figure 8:**
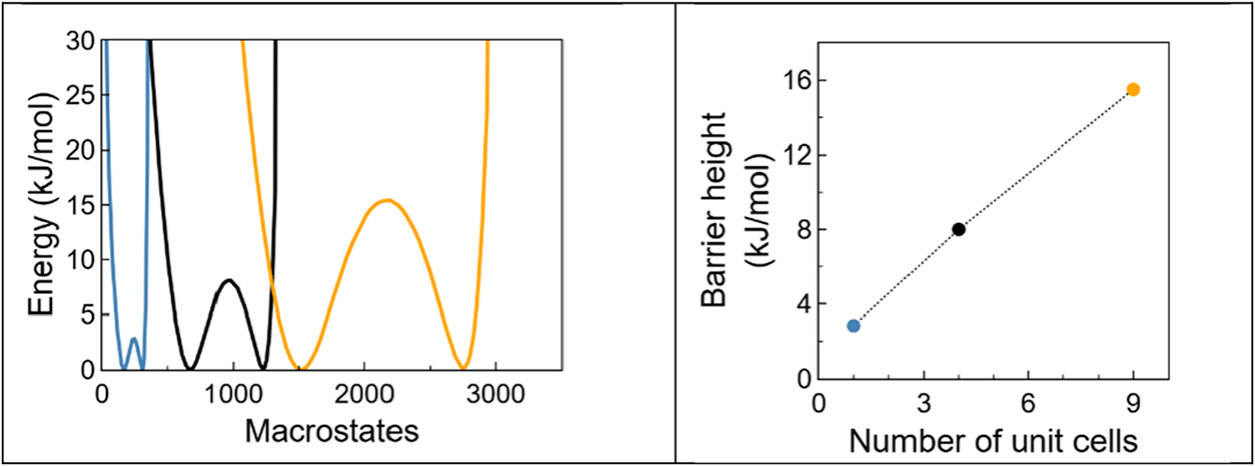
Left: Free energy profiles for CH_4_ adsorption in IRMOF-8 at *T* = 110 K and *P* = 1385 Pa, simulated using MC boxes composed of (1 × 1 × 1 in blue), (2 × 2 × 1 in black), and (3 × 3 × 1 in yellow) IRMOF-1′s unit cells. The free energy barriers height (right panel) is shown as a function of the number of unit cells included in the MC simulation box. Instantaneous snapshots of fluctuations in a 3 × 3 × 1 unit cell are presented in SI, Figure S7.

Interestingly, increasing the size of the simulation box alone does not lead to the emergence of additional intermediate metastable states (i.e., additional local minima) in the free energy landscape of CH_4_ adsorption. This observation supports the interpretation of the transformations as a discontinuous, first-order process, in which intermediate macrostates represent weighted (statistical) averages over the two coexisting metastable microscopic states, corresponding to either fully filled or fully empty pores. A similar conclusion was reached in our previous study on CH_4_ adsorption in IRMOFs.^8^ These results reinforce the hypothesis that pore filling proceeds via a first-order transformation, independent of the Monte Carlo simulation box size. At the same time, they show that the metastability observed in this system originates from a mechanism fundamentally different than that reported for water adsorption,^14–19^ where the metastable states originate from the formation and rearrangement of molecular clusters. In the case of water, the number of metastable states may depend on the size of the simulated sample (MC box), and their presence is associated with extremely slow equilibration dynamics.^16,18,19^

### Origin of the Metastability

A fundamental question in the study of adsorption is the origin of metastability. Simulations of CO_2_ adsorption reveal that at low temperatures, certain macrostates are effectively inaccessible, neither thermodynamically stable nor kinetically reachable. Rather than stabilizing in a single well-defined macrostate (which would correspond to lower entropy), the system establishes a dynamic equilibrium between two states, each statistically populated over time. These states are separated by a finite free energy barrier, making the probability of transitions between the states dependent on temperature.

Pore symmetry plays a crucial role in governing adsorption behavior. At low temperatures, the symmetry of the adsorption sites distribution is reflected in the symmetry of the adsorbed structure. Once the maximum capacity of these sites is reached, the adsorbed phase must be restructured to accommodate additional molecules. This restructuring can proceed discontinuously, particularly in highly symmetrical pores. Adsorbate configurations that preserve the symmetry of the framework have often lower energy and thus are thermodynamically more favored. In contrast, intermediate macrostates that break this symmetry tend to be both higher in energy and lower in entropy, which renders them thermodynamically inaccessible. As a result, the system avoids these asymmetric configurations, and metastability emerges because of the combined enthalpic and entropic penalties associated with symmetry-breaking intermediate states.

At higher temperatures, the behavior of the system changes. Increased thermal energy introduces greater disorder into the adsorbed phase, enhancing the entropy and allowing the system to explore a broader region of configuration space. As a result, the free energy barriers separating macrostates become easier to overcome (or disappear), and the adsorption process becomes more continuous in nature.

A complementary perspective emerges from the analysis of CH_4_ adsorption, where additional metastable states are observed, particularly at low adsorbate densities. In this case, the spherical shape of the methane molecule and the symmetric distribution of adsorption sites within the pore play a critical role. In our previous work,^9^ we identified a distinct metastable configuration that disappeared at elevated temperatures. This structure was stabilized by the geometric symmetry of the pore walls and corresponded to a low-density gas molecules arrangement aligned with shallow adsorption sites (Figure 9). At very low loading, methane molecules preferentially populate these shallow, yet energetically favorable, sites.

**Figure 9:**
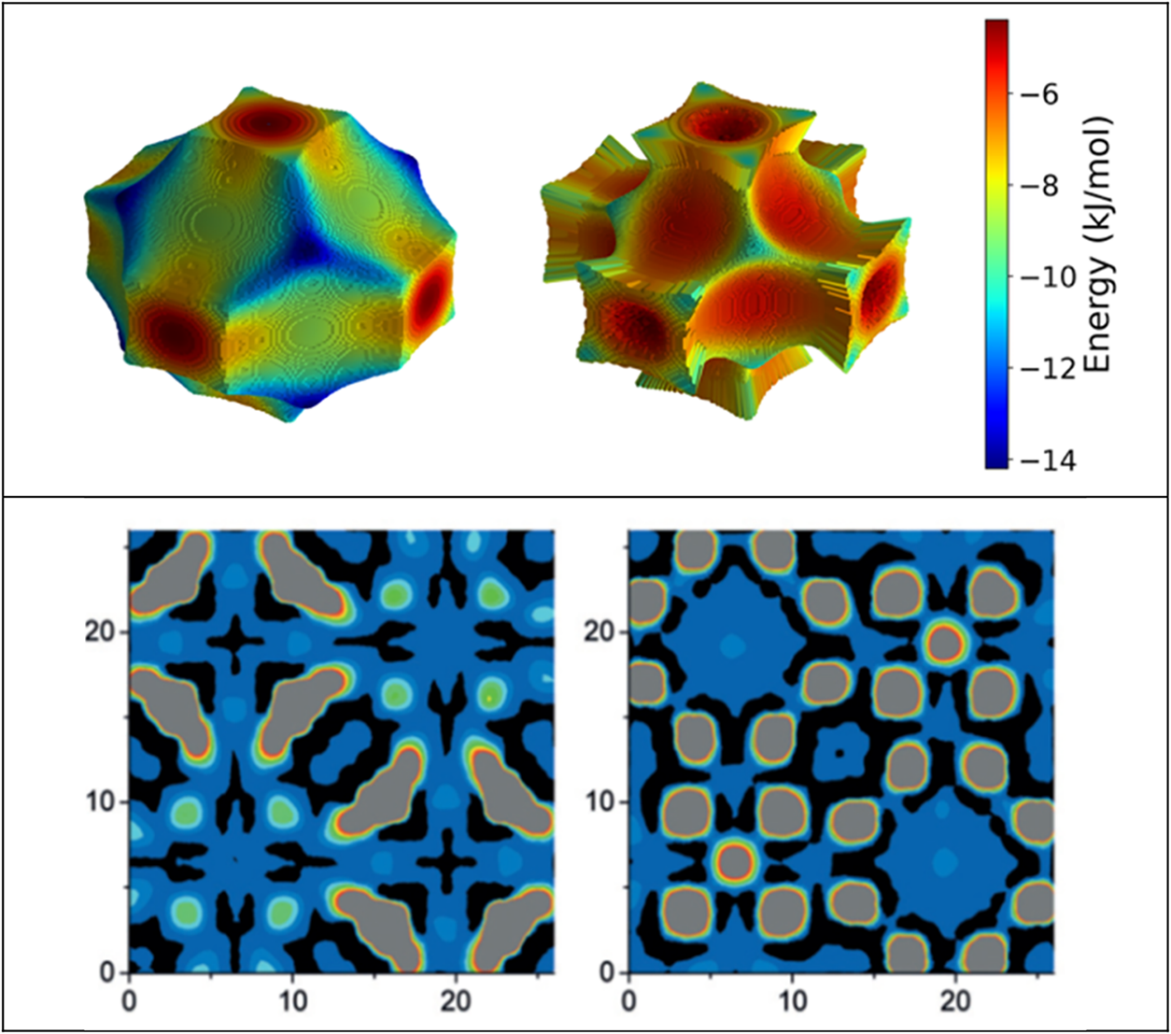
Adsorption energy distributions and spatial distribution of CH_4_ in IRMOF-1. Two upper Figures: adsorption energy maps for CH_4_ in the smaller (left) and larger (right) pore of IRMOF-1. The strongest adsorption sites are in the corners of the larger pore. Lower Figures: methane distribution at low uptake (left), closely matching the geometry of the most favorable adsorption sites and methane distribution at higher loading (right) where CH_4_ density distribution becomes more localized due to packing effects and enhanced molecule–pore interactions. Gray areas represent methane density.

As the adsorbate density increased, the distribution of CH_4_ molecules became more localized, giving rise to a configuration that was also symmetric, but distinct from the low-density state. This newly formed structure, though metastable, was stabilized by the interplay of molecule–pore interactions and packing constraints. It occupied a region of the free energy landscape near the barrier separating macrostates, illustrating how geometric symmetry and molecular shape contribute to the emergence and persistence of metastable states in adsorption processes.

In summary, at low temperatures and within highly symmetric pores, the emergence of two states corresponding to low- and high-density states appears to be a natural consequence of the system’s energetic and geometric constraints. At low adsorbate densities, the ordered pore environment energetically favors only symmetric molecular arrangements. As density increases, the balance between the gas–gas and gas-pore interactions begins to influence the stability and structure of the final configuration. With increasing temperature, thermal energy introduces disorder and raises the system’s entropy, enabling stabilization of a broader ensemble of macrostates. Additional metastable states may arise when specific distributions of adsorption sites provide sufficient energetic stabilization (see also Figure S6).

A key advantage of molecular simulation is the ability to model water adsorption in frameworks that are experimentally inaccessible due to their structural instability in aqueous environments. Accordingly, we compare the metastability observed in CH_4_ and CO_2_ adsorption with that of water in micropores of IRMOF-1, IRMOF-8 and Al­(OH)­(1,4-ndc) MOF,^20^ where strong water–water interactions play a leading role. These interactions have been reported to promote multiple free energy minima,^14–16^ corresponding to water clusters that assemble within the pores into energetically stable networks resembling the structure of bulk liquid water.^16^ In Al­(OH)­(1,4-ndc) MOF, these macrostates represent metastable configurations of the system (see Figure 10). The number of free energy minima increases with the size of the Monte Carlo simulation box, suggesting that in real (macroscopic) systems, the transition between the low- and high-density states of adsorbed water may be continuous. In the thermodynamic limit, where system size approaches infinity, the number of metastable minima likewise becomes infinite.^14^ In this regime, water adsorption proceeds via sequential filling of pores, with each occupancy level corresponding to a distinct metastable state. This behavior is consistent with the previously reported simulation studies.^14–16^ At the same time, water adsorption in IRMOFs structure leads to free energy form like the one observed for CO_2_, with no evidence of cluster formation (see Figure 10). Finally, to assess whether pore connectivity could facilitate cluster formation, we simulated CH_4_ and CO_2_ adsorption in the Al­(OH)­(1,4-ndc) MOF, which features disconnected one-dimensional channels. No evidence of clustering was observed; adsorption of both CH_4_ and CO_2_ was uniform throughout the pores. These results highlight the need for further investigation to understand how different ordered crystalline pore structures influence water adsorption mechanisms.

**Figure 10:**
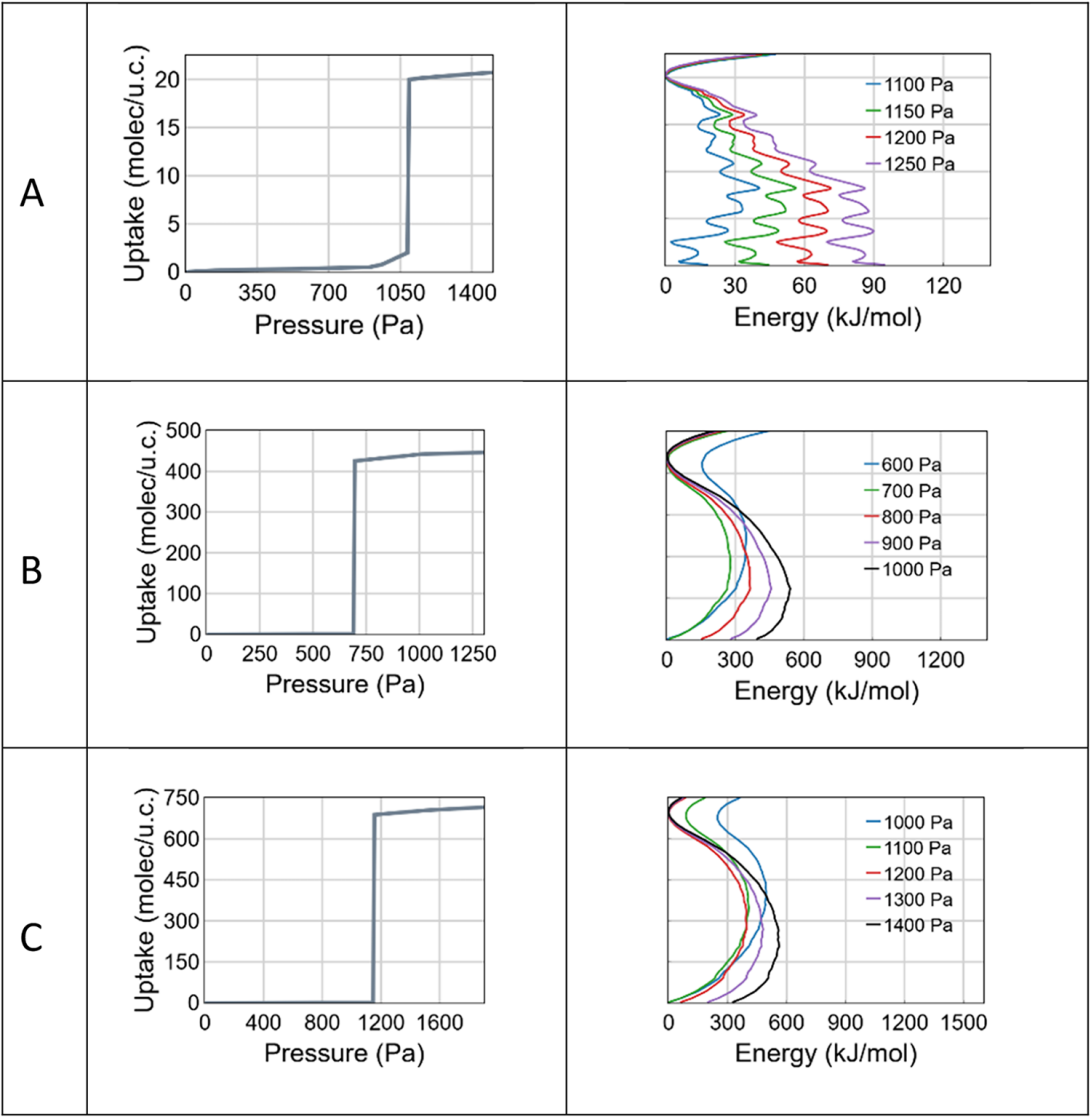
Isotherms and free energies profiles (*T* = 298 K) of water adsorption in (A) Al­(OH)­(1,4-ndc), (B) IRMOF-1 and (C) IRMOF-8 modeled using the TIP4P-Ew water potential.

The nature of metastability in water adsorption differs from that observed for CO_2_ and CH_4_ in IRMOF structures. Water clusters are stabilized by strong intermolecular hydrogen bonding, resulting in deep energy minima. This makes the addition or removal of individual molecules energetically unfavorable, leading to discrete cooperative adsorption events. Such a collective behavior is absent in the gas-like adsorption of CO_2_ and CH_4_. Consequently, water fills the pores sequentially, each step corresponding to the formation of a stable cluster. The number of resulting metastable states scales with the system size.

In contrast, for CO_2_ and CH_4_ in IRMOFs, intermediate metastable states emerge only under specific symmetry conditions and are stabilized by the delicate competition between fluid–fluid and fluid–framework interactions. These states are typically observed at low temperatures and in highly ordered pore environments. In the absence of such symmetry constraints or when thermal energy introduces a structural disorder, these intermediate, partially filled configuration states become thermodynamically unstable, and no metastable states persist. The systems then transition smoothly between low- and high-density phases, resulting in continuous adsorption behavior.

In conclusion, water represents a unique case among adsorbates due to its pronounced hydrogen bonding, which results in strong, directional electrostatic interactions. These interactions substantially slow down adsorption kinetics and promote the formation of stable molecular clusters within the pores. In narrow or hydrophilic pores, water often undergoes phase-like transitions (such as pore filling or emptying) that result in metastable states with long residence times, posing significant challenges for numerical simulations.

For the same reasons, water adsorption is often cooperative: the initial adsorption of a few water molecules reduces the energetic barriers for subsequent uptake. Furthermore, confinement imposes substantial constraints on both rotational and translational degrees of freedom, leading to notable entropy losses. These constraints, in combination with directional bonding, contribute to the complexity and distinct behavior of water under nanoconfinement.

Table 2 below provides a comparative summary of the key features associated with the adsorption and metastability of water and CO_2_/CH_4_ in crystalline microporous materials.

**Table 2:** Key Differences between the Mechanisms of Adsorption: Water versus CO_2_/CH_4_

feature	water	CO_2_/CH_4_
intermolecular interactions	strong, directional hydrogen bonds, which drive cluster formation inside pores	weak, nondirectional van der Waals or quadrupolar interactions
adsorption behavior	cooperative adsorption: once a few molecules adsorb, it becomes energetically favorable for more to follow rapidly	gradual, smoother isotherms with sharp transitions only at temperature close to the triple point temperature
free energy surface	rugged, with deep multiminima	smooth and continuous, with a limited number of minima
GCMC acceptance rate	often very low: inserting new molecules into a dense, hydrogen-bonded cluster has a very low acceptance rate	moderate to high: insertion energies fluctuate mildly, and moves are more often accepted
metastability	pronounced (e.g., pore filling): it leads to metastable states with long residence times	only at very low temperature (below the triple point)
sampling bottlenecks	severe	only at very low temperature (below the triple point)

## Discussion

Metastability plays a fundamental role in molecular simulations of adsorption, particularly in systems characterized by complex energy landscapes or undergoing rare events such as phase transitions. In this study, we examined the adsorption mechanisms of CO_2_, CH_4_, and H_2_O in crystalline microporous materials, with a focus on how metastable states influence dynamic equilibrium and sorption behavior. We show that the presence of free energy barriers, separating the metastable states, can significantly hinder transitions within simulation-accessible time scales. These barriers, and the corresponding rare fluctuations, are key to understanding adsorption in highly structured porous materials, where conventional interpretations based on equilibrium thermodynamics may be insufficient.

The TMMC approach to free energy calculations has an intrinsic limitation coming from its algorithm, which only accounts for fluctuations between the neighboring states (Δ*N* = 1). As a result, it does not capture stable situations in which fluctuations occur between different distant TMMC states. For this reason, TMMC free energy calculations must be always complemented by GCMC analysis of fluctuations accessible in the studied system. When such fluctuations are possible, the TMMC free energy does not necessarily represent the total free energy of the system.

While many microporous materials display IUPAC Type I isotherms, this classification oversimplifies the behavior observed in highly ordered frameworks.^2^ Our simulations reveal that in crystalline micropores, particularly near the adsorbate’s triple point, adsorption proceeds through a low-density phase followed by a sharp pore-filling transition. As the temperature increases, this behavior shifts toward smoother, Type V-like isotherms.

The ordered geometry of micropores, in combination with framework symmetry and fluid–fluid interactions, gives rise to complex dynamics and unexpected metastable behavior. In the case of CH_4_ and CO_2_ adsorption, we observed pronounced fluctuations between high- and low-density pore-filling states, governed by the height of the free energy barriers separating them. For CO_2_ in IRMOF-1, strong intermolecular CO_2_–CO_2_ interactions and the high pore symmetry stabilize two distinct adsorption states, effectively suppressing intermediate configurations. In contrast, CH_4_ exhibits transitions between competing low-density states, influenced by the spatial distribution of adsorption sites and the underlying framework symmetry.^9^

Water adsorption represents an even more intricate case.^14–17,21–23^ Unlike simple gases, water’s behavior is governed by directional hydrogen bonding, which promotes the formation of stable molecular clusters and results in multiple distinct free energy minima. These metastable states arise from strong electrostatic interactions and lead to exceptionally slow equilibration in simulations, especially at low temperatures. Therefore, conventional grand canonical Monte Carlo approaches often fail to fully capture the adsorption process, resulting in hysteresis and under-sampling of relevant configurations.

Understanding metastable mechanisms is crucial for the rational design of materials for applications such as water harvesting and thermal energy storage. Yet accurately simulating water adsorption under confinement remains a major challenge. No existing force field fully captures all of water’s essential properties, particularly hydrogen bonding, polarizability, and molecular flexibility, when restricted to a nanoscale environment. Furthermore, the intrinsically rugged free energy landscape significantly hampers sampling efficiency and imposes computational cost.

## Conclusions

The present study highlights the pivotal role of metastability in governing adsorption processes under confinement. Our findings demonstrate how pore geometry, fluid–fluid and fluid–framework interactions, and temperature jointly shape sorption dynamics across different adsorbates. To achieve realistic and predictive modeling of these systems, particularly for complex molecules like water, future efforts must explicitly account for metastability through advanced sampling techniques and the development of more accurate, physically grounded force fields. These improvements are essential to ensure the rational design of nanoporous materials for practical applications.

It is noteworthy that an adsorption mechanism similar to this observed in IRMOFs has also been reported in a simplified model of ordered pores.^24,25^ This underscores the need for further in-depth investigations to fully understand the role of metastability in the adsorption process across both realistic and idealized systems. By its nature, metastability is the field of research that must be strongly assisted and supported by numerical modeling, using methodologies that allow one to calculate free energy of the studied systems.

In summary, this manuscript identifies a previously unrecognized adsorption mechanism in crystalline microporous materials, arising from nanoconfinement rather than from classical capillary condensation. Using transition-matrix Monte Carlo simulations, we demonstrate that micropore filling can proceed via equilibrium coexistence of structurally distinct adsorption states within a single pore, leading to multimodal equilibrium distributions and large-amplitude fluctuations even in the absence of kinetic trapping. This behavior is fundamentally different from metastability associated with hysteresis in mesoporous systems and has not been reported previously. The work further shows that conventional free-energy profiles may be insufficient to characterize adsorption equilibria under such conditions, motivating a physically grounded methodological recommendation to complement free-energy calculations with fluctuation analysis. These findings advance the conceptual understanding of fluid behavior under nanoconfinement and are broadly relevant to nanoporous materials studied across nanoscience, energy, and environmental applications.

## Methods

### Experimental setup

#### Synthesis

IRMOF-1 was prepared in N,N-diethylformamide (DEF) solution following the procedure described in the literature.^26^ Due to the absence of product after the initially postulated 8 h reaction time, the synthesis duration was extended to a total of 24 h. All reagents and solvents were of analytical grade (Sigma-Aldrich, TokyoChemical Industry) and were used as received, without further purification.

#### Powder X-ray Diffraction (PXRD) Measurement

The PXRD pattern of desolvated IRMOF-1 was recorded at room temperature using a STOE STADI P diffractometer equipped with Cu–Kα_1_ radiation (λ = 1.54059 Å) and a 1D Mythen detector (Dectris). Data were collected in transmission mode using a rotating flatbed sample holder. The measurements were performed using a steps size of 6° in 2Θ and an exposure time of 20 s per step. Prior to measurement, IRMOF-1 was mounded in the glovebox and sealed under parafilm to prevent exposure to air.

#### Gas Adsorption Measurements

Prior to the physisorption measurements, the as-synthesized IRMOF-1 was washed eight times with 20–30 mL of anhydrous DMF, allowing the solid soak for 1–3 h during each washing step. Subsequently, the DMF was decanted, and the sample was washed seven times with 20–30 mL of anhydrous CH_2_Cl_2_, again with 1–3 soak intervals per cycle. Following solvent exchange, the sample was dried using the Schlenk technique under a flow of Argon to remove the excess of solvent. Final desolvation was performed under reduced pressure (∼10^–3^ kPa) at 363 K for ∼16 h. The desolvated IRMOF-1 was then transferred in the Schlenk tube into glovebox (MBRAUN) under an inert atmosphere for storage and handling.

Gas adsorption measurements for CO_2_ (99.999% purity) and CH_4_ (99.999% purity) were conducted using a *BELSORP-max* adsorption apparatus (MicrotracBEL Corp.).

*BELSORP-max* was connected to the home-built adsorption cell, connected to the closed cycle helium cryostat DE-202AG (ARS). The adsorption temperature was precisely controlled using LS-336 temperature controller (LAKE SHORE), and the excess heat generated by the cryostat was dissipated via water-cooled helium compressor ARS-2HW.

For each experiment 32.2 mg of desolvated IRMOF-1 was loaded into the adsorption cell, which was sealed externally with a copper dome using a copper gasket. The system was thermally isolated under dynamic vacuum (*p* < 10^–4^ kPa) and connected to the *BELSORP-max* adsorption instrument using a 1/8 in. stainless steel capillary. Following assembly, the sample was degassed under a dynamic ultrahigh vacuum of 0.01 Pa at 298 K for 12 h to ensure complete removal of residual adsorbates. Adsorption equilibrium was defined as the conditions under which pressure variations remained within 1% over a period of 500 s.

### Numerical Setup

#### Methodology of free energy Calculations—Transition Matrix Grand Canonical Monte Carlo (TM-GCMC)

The free energy profile, Ω­(*N*), where *N* denotes the number of adsorbed molecules, is a fundamental descriptor for identifying true thermodynamic equilibrium states, metastable configurations, and assessing the overall metastability of a system. However, this profile (Figure 1, central panels) cannot be directly obtained from the conventional Metropolis grand canonical Monte Carlo (GCMC) simulations, as the method’s inherent limitations prevent adequate exploration of the full configuration space. To overcome this, the Transition Matrix Monte Carlo (TMMC), has been successfully employed to reconstruct the adsorption free energy landscape. This approach provides valuable insight into both equilibrium and nonequilibrium aspects of the adsorption process. Moreover, kinetic information can be inferred from the energy barriers separating local minima of Ω­(*N*), to estimate transition rates between metastable and stable states.

The fundamental principles of the Transition Matrix Grand Canonical Monte Carlo (TM-GCMC) method are extensively documented in the literature^4,18,19^ and are described in detail in our previous paper.^8^ This advanced simulation technique offers enhanced sampling efficiency compared to conventional GCMC methods and enables the calculations of free energy profiles as a function of the number of system’s macroscopic states quantified by the number of adsorbed molecules *N*, at specified external pressure or chemical potential.

#### Methodology of Adsorption Calculations—Grand Canonical Monte Carlo (GCMC)

GCMC simulations were carried out to calculate both the adsorption and fluctuations using RASPA software. A 2 × 2 × 2 supercell was used in the simulations to minimize the influence of periodic boundary conditions. The molecules were rigid. Translation, reorientations, insertion/deletion moves were chosen randomly with equal probability. We initially used 1 million equilibration MC cycles for all runs. A cycle contains N MC steps, where N is the number of adsorbate molecules in the system at the beginning of the cycle (but not less than 20).

## Supplementary Material



## Notes

Structure model and force field, CO_2_ numerical isotherms, fluctuations and free energy, Distribution of density, Experimental setup (PDF)
