# Supplementary material for: Adsorption Mechanism in Crystalline Micropores: Multimodal Fluctuations, Metastability and Phase Transformations in Nanoconfinement
Source: ACS Nano. 2026 Jan 27;20(5):4204–16. doi: 10.1021/acsnano.5c16606 (PMC12895509; doi:10.1021/acsnano.5c16606)
Supplement: Supplementary file 1 [file nn5c16606_si_001.pdf]

Supplementary Information

Adsorption mechanism in crystalline micropores: multimodal fluctuations, metastability and phase transformations in nanoconfinement

Małgorzata Stankiewicz[1], Anthony Dorhauer,[2,3] Kornel Roztocki[4], Volodymyr Bon[5], Bartosz Mazur[1], Carlos Wexler, [2,3] Stefan Kaskel[5], Lucyna Firlej[1,2,6*], Bogdan Kuchta[1,2,7*]

1. Faculty of Chemistry, Wroclaw University of Science and Technology, Wybrzeze Wyspianskiego 27, 50-370 Wroclaw, Poland
2. Department of Physics and Astronomy, University of Missouri, Columbia, MO 65211, USA
3. Materials Science and Engineering Institute, University of Missouri, Columbia, MO 65211, USA
4. Faculty of Chemistry, Adam Mickiewicz University, Uniwersytetu Poznańskiego 8, 61-614 Poznań, Poland
5. Department of Inorganic Chemistry, Faculty of Chemistry and Food Chemistry, Dresden University of Technology; Dresden, 10169, Germany
6. Laboratory Charles Coulomb, UMR CNRS 5221, University of Montpellier, 34095 Montpellier, France
7. Laboratory MADIREL, Aix-Marseille University – CNRS, 13013 Marseille, France

Table of contents

1. **Structure model and force field 1**
2. **CO_2 numerical isotherms, fluctuations and free energy.. 4**
   - **IRMOF-8/CO2**
   - **IRMOF-10/CO2**
   - **IRMOF-14/CO2**
3. **Distribution of density 7**
4. **Instantaneous snapshots of fluctuations in a 3×3×1 Unit Cell 8**
5. **Experimental setup and AIF files... 9**

Structure model and force field

The crystal structure of IRMOF-1 was obtained from the work of Eddaoudi[1] et al., with the corresponding CIF file available in the RASPA[2] simulation package. During all simulations, the framework was treated as rigid. Interactions between the framework and the adsorbate (CO_2 and methane) were modeled using the standard Lennard-Jones (LJ) 6-12 model. The LJ parameters for the MOF and adsorbate atoms (listed in **Table S1**) were taken from the DREIDING[3], UFF[4], and TraPPE[5] force fields. Charges were estimated using the PACMOF Python library — a pre-trained Random Forest model that delivers DDEC-level accuracy for MOF atomic charges at high throughput[6]. All interactions were truncated at a cutoff value of 14.0 Å (12.8 Å for IRMOF-1); tail corrections were applied only to gas–gas interactions. Lorentz–Berthelot mixing rules were applied for the MOF–adsorbate cross terms. The fugacity coefficient was calculated using the Peng-Robinson equation of state, as implemented in the RASPA software. The atom types in

the IRMOF structures are defined as follows: Zn is the metal center; O1 is the central oxide ion in the Zn_4O cluster; O2 is the carboxylate oxygen from the linker coordinating to Zn. For IRMOF-1, C1 is the carboxylate carbon; C2 is the aromatic carbon in the para position relative to the carboxylate group; and C3 is the aromatic carbon in the meta position. Atom types in IRMOF-8, IRMOF-10, and IRMOF-14 are illustrated in Fig. S2.

Table S1. Lennard-Jones and electrostatic parameters used in GCMC simulations.

Species	ε (K)	σ (Å)	Charge IRMOF-1	Charge IRMOF-8	Charge IRMOF-10	Charge IRMOF-14
Zn	62.4	2.462	0.973	0.996	0.929	0.907
O1	48.158	3.033	-1.036	-1.05	-1.063	-1.094
O2	48.158	3.033	-0.541	-0.567	-0.56	-0.58
C1	47.857	3.473	0.591	0.615	0.595	0.605
C2	47.857	3.473	-0.018	-0.035	-0.024	-0.02
C3	47.857	3.473	-0.112	-0.138	-0.121	-0.142
C4	47.857	3.473	-	-0.12	-0.121	0.095
C5	47.857	3.473	-	-0.12	0.08	0.023
C6	47.857	3.473	-	0.089	-	-0.121
H1	7.649	2.846	0.128	0.118	0.131	0.117
H2	7.649	2.846	-	-	0.124	0.115
C (CO2)	27	2.8	0.7	0.7	0.7	0.7
O (CO2)	79	3.05	-0.35	-0.35	-0.35	-0.35
CH4	148	3.73	-	0	-	-

Table S2. Force Field Parameter Comparison for CO_2 Adsorption in IRMOF-1 at 218 K.

	Current work			Previous work			Datar et. al.			Walton et. al.		
Atom type/ parameter	ε (K)	σ (Å)	LJ treatment	ε (K)	σ (Å)	LJ treatment	ε (K)	σ (Å)	LJ treatment	ε (K)	σ (Å)	LJ treatment
Zn	62.4	2.462	truncated with no tail correction	27.68	4.05	truncated with no tail correction	27.69	4.04	shifted	0.42	2.7	truncated with no tail correction
O1	48.158	3.033		48.158	3.033		48.18	3.03		700	2.98	
O2	48.158	3.033		48.158	3.033		48.18	3.03		70.5	3.11	
C1	47.857	3.473		47.86	3.4		47.86	3.37		47	3.74	
C2	47.857	3.473		47.86	3.4		47.86	3.37		47.86	3.47	
C3	47.857	3.473		47.86	3.4		47.86	3.37		47.86	3.47	
H	7.649	2.846		7.65	2.85		7.85	2.85		7.65	2.85	
C_CO2	27	2.8	truncated with tail correction	27	2.8		28.129	2.757		27	2.8	
O_CO2	79	3.05		79	3.05		80.567	3.033		79	3.05	

Sensitivity of GCMC simulations to interaction potentials

In GCMC simulations of adsorption, the choice of interaction potentials is critical for producing accurate and reliable predictions. These potentials define how adsorbate molecules interact with both the porous network and with each other. Even minor adjustments to key parameters such as Lennard-Jones (LJ) well depth (ε) and size parameter (σ), truncation/shift schemes, or atomic charges can lead to significant changes in adsorption isotherms. For instance, changes in LJ parameters directly influence the depth and position of the potential well, thereby modifying the interaction strength and the adsorption energy

landscape. Adjustments to truncation and shifting rules further affect long-range interactions: "shifted" potentials ensure a smooth decay to zero at the cutoff, while "truncated" potentials may underestimate attractive forces near the cutoff radius, especially in larger pores. Electrostatic interactions are highly sensitive to assigned partial charges, especially for polar molecules like CO_2. The charge distribution on atoms like C_CO_2 and O_CO_2 influences not only the strength of the electrostatic component but also the spatial distribution of adsorbed molecules. These factors collectively affect the balance between adsorption and desorption across a range of pressures. In our simulations, noticeable shifts in adsorption isotherms were observed when comparing different parameter sets (our modified potential, those reported by Walton et al. (2007, adapted for use in RASPA), and those used by Datar, as well as in our earlier work (see **Table S2**) [7-10]. Even small changes in LJ parameters, charge assignments, or truncation rules led to clear differences in isotherms. This highlights the importance of careful parameters and validation against experimental data. Our observations are consistent with previous studies, which have demonstrated that adjusting force field parameters can substantially modify the adsorption behavior; for example, changes in LJ parameters and charge assignment have been shown to influence adsorption in MOFs.[7,9,10] These findings reinforce the need for systematic evaluation and validation of interaction potentials to ensure that GCMC simulations yield quantitatively accurate and physically meaningful results.

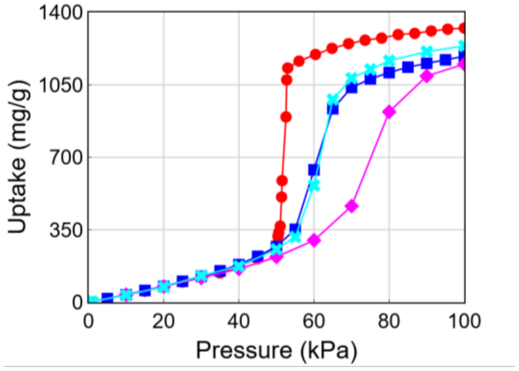

Figure S1. CO_2 isotherms in IRMOF-1 simulated at 218 K using 4 different Force Field parameters. Red circles: force field used in the current study. Blue squares: force field used in a previous study[10]. Magenta diamonds: data from Datar et al.[9] Cyan crosses force field used by Walton et al.[7]

IRMOFs structures

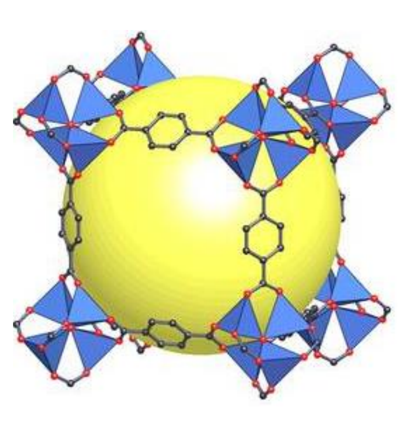	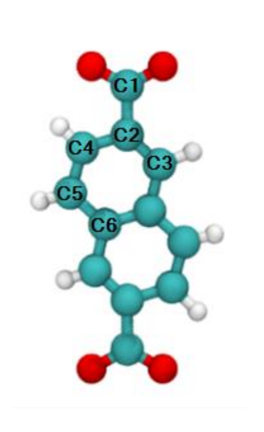	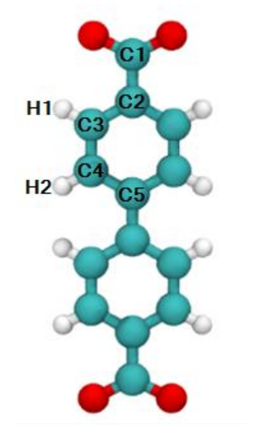	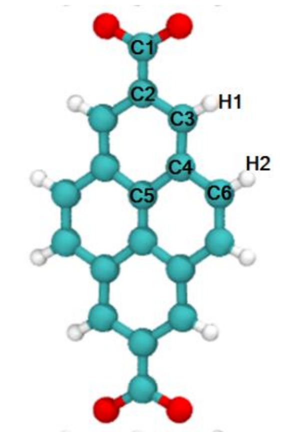
IRMOF-1 (a = 25.832 Å)	IRMOF-8 (a = 30.092 Å)	IRMOF-10 (a = 34.281 Å)	IRMOF-14 (a = 34.381 Å)

Figure S2. The IRMOF-X (X = 1,8,10,14) unit cell structures.

CO_2 numerical isotherms, fluctuations and free energy.

IRMOF-8/CO2

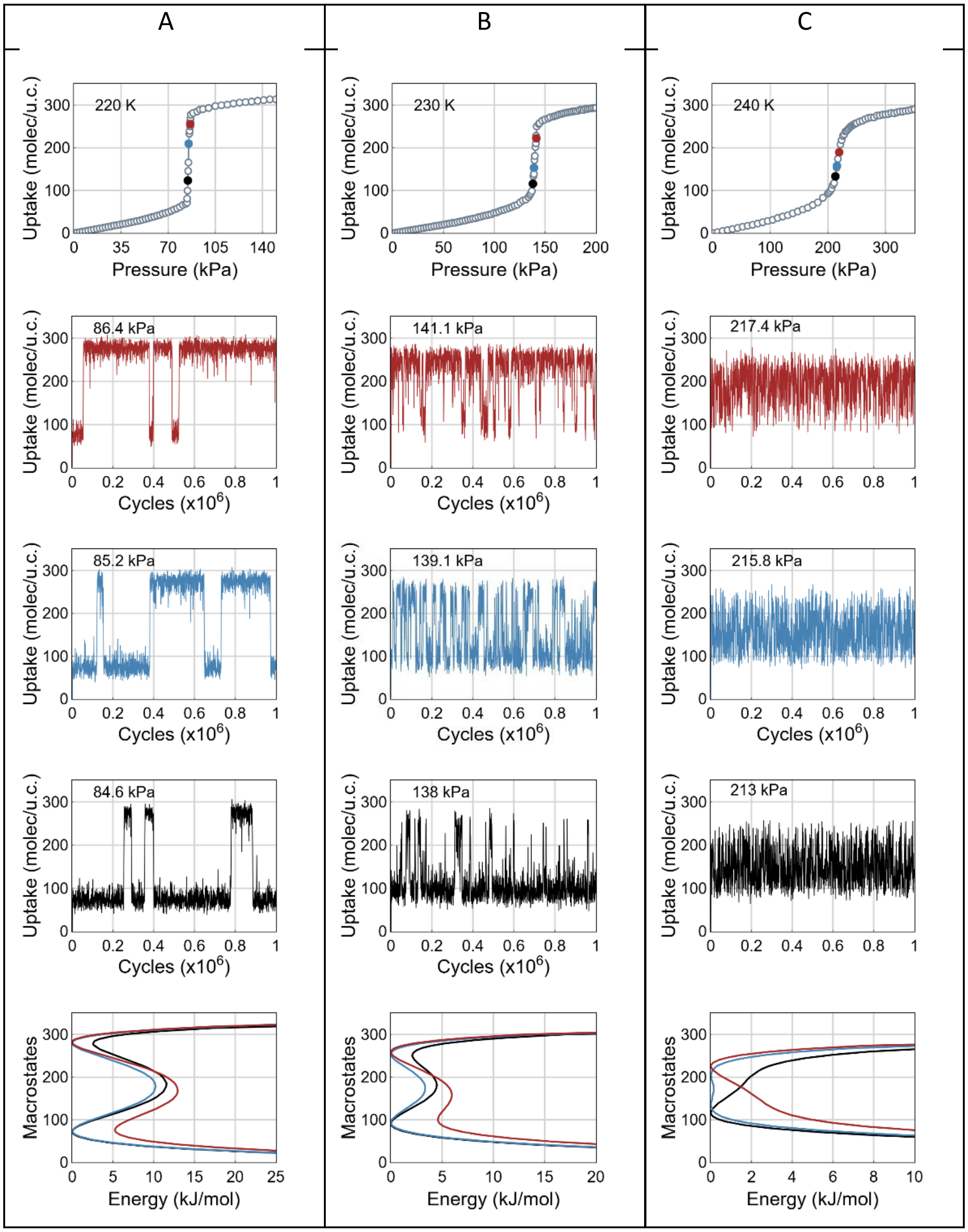

Figure S3. Free energies and the corresponding bimodal fluctuations of the CO_2 uptake in IRMOF-8 at T=220 K and 230 K, and 240 K.

IRMOF-10/CO2

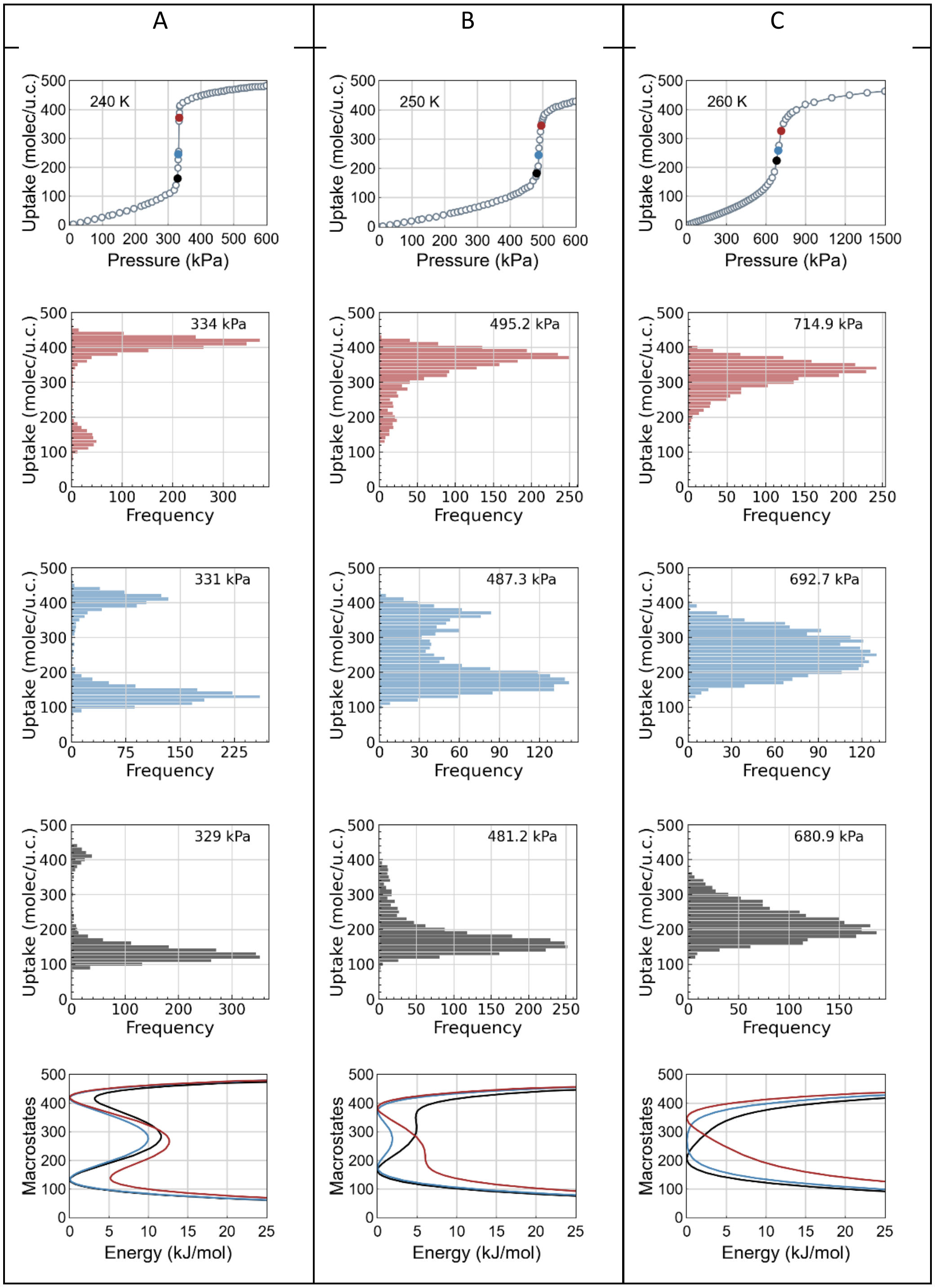

Figure S4. Free energies and the corresponding bimodal fluctuations of the CO_2 uptake in IRMOF-10 at T=240 K and 250 K, and 260 K. The fluctuations are not shown explicitly, only the distributions of the number of adsorbed molecules are shown.

IRMOF-14/CO2

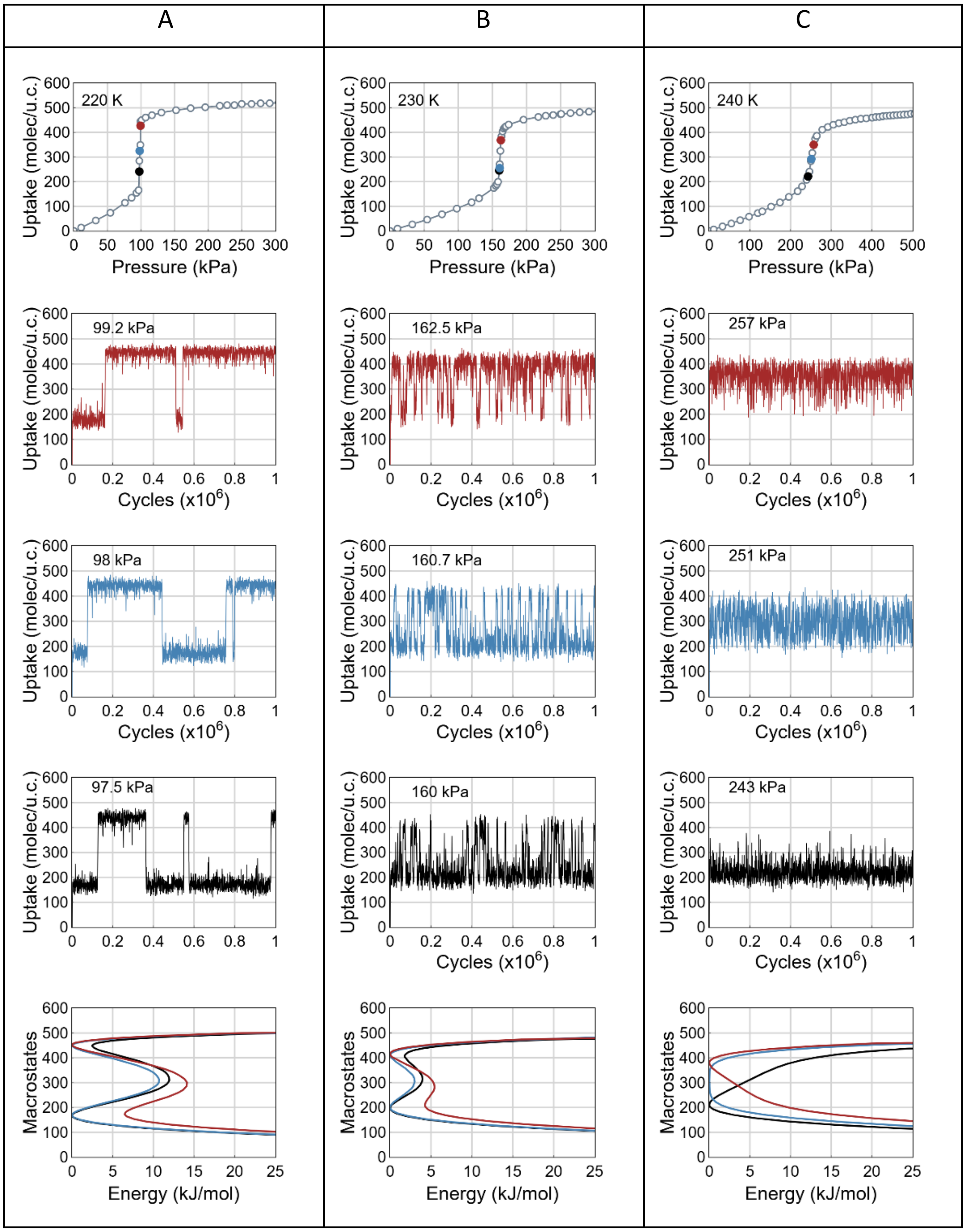

Figure S5. Free energy and the corresponding bimodal fluctuations of the CO_2 uptake in IRMOF-14 at T=220 K and 230 K and 240 K.

Data for the simulation isotherms can be found in https://github.com/mstanki

Distribution of density

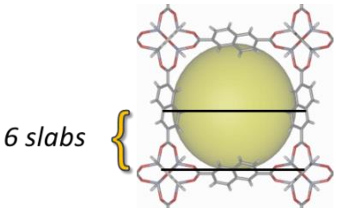

A.

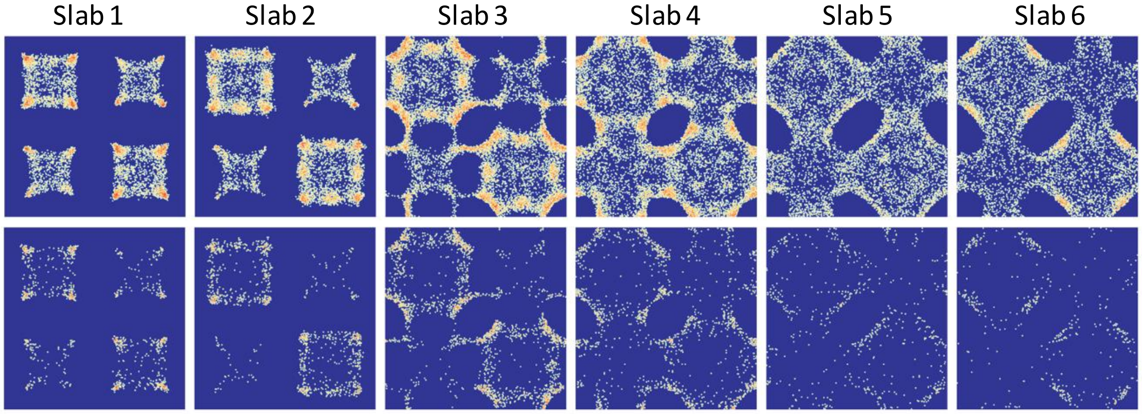

B.

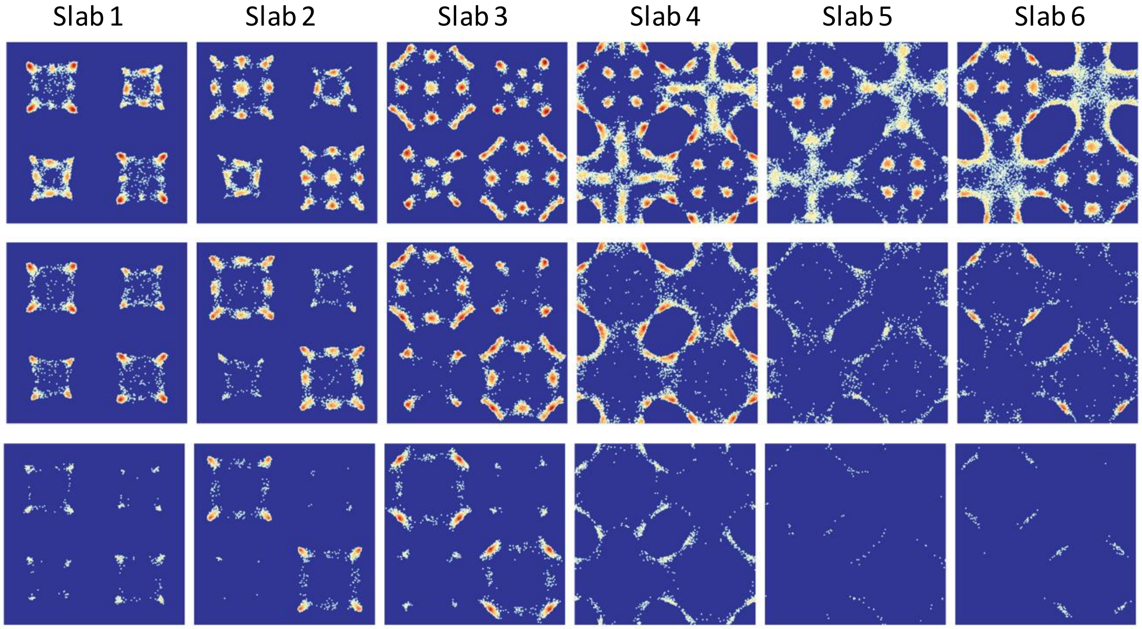

C.

Figure S6. (A) Schematic representation of the IRMOF-8 pore, divided into 6 horizontal slabs across 4 adjacent pores. Slab 1 is positioned closest to the metal nodes, while Slab 6 lies near the center of the pore. This segmentation facilitates the analysis of vertical density variations during adsorption; (B) CO_2

adsorption in IRMOF-8 at 230 K at two selected pressures. The bottom six panels show the CO_2 density distribution at 100 kPa (low-pressure regime prior to the adsorption step), while the top six panels correspond to 139.1 kPa (midpoint of the adsorption step, see Figure 3SB). (C) CH_4 adsorption in IRMOF-8 at 115 K at three selected pressures. The bottom row shows the CH_4 density distribution at 9.79 kPa (low-pressure state), the middle row at 19.9 kPa (onset of the step), and the top row at 20.5 kPa (upper part of the step). These states correspond to the orange, blue, and red points on the isotherm and the corresponding free energy profiles (Figure 6A).

Instantaneous snapshots of fluctuations in a 3×3×1 Unit Cell

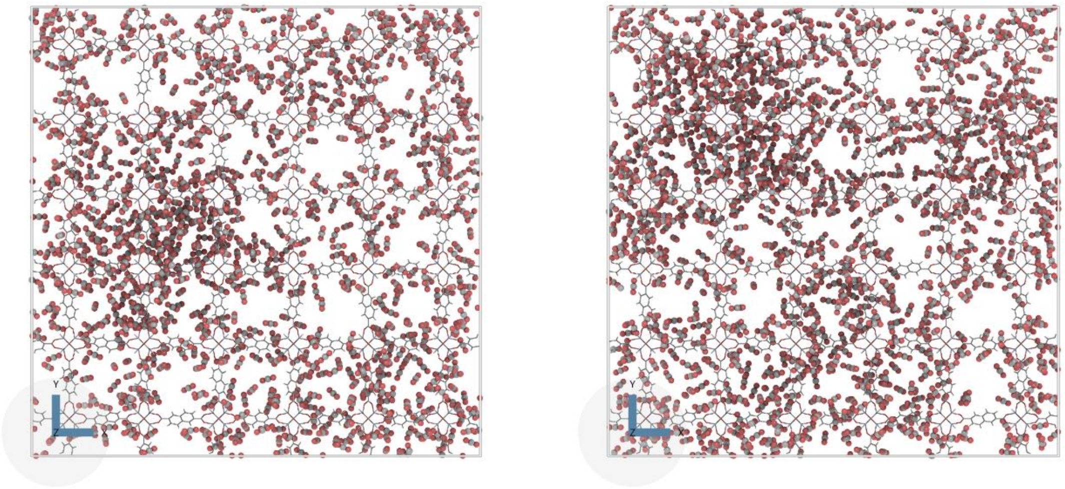

A.

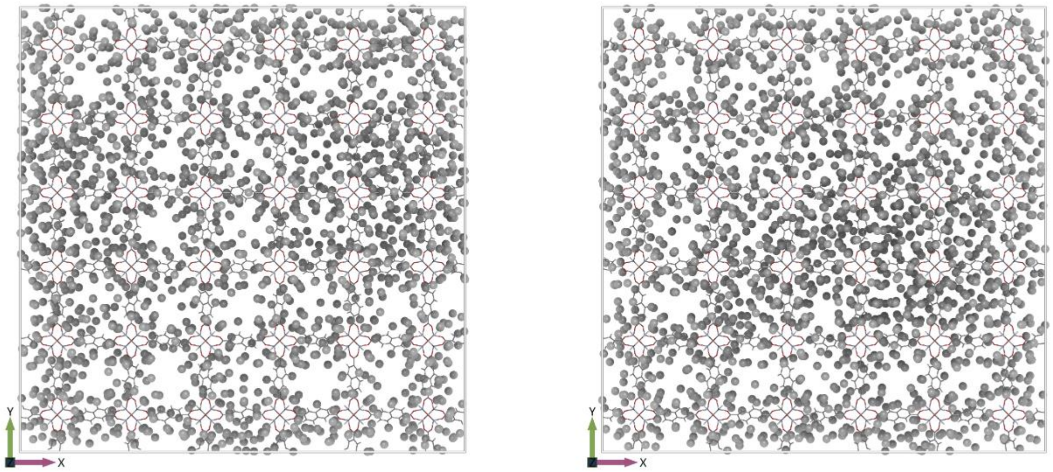

B.

Figure S7. (A) CO_2 adsorption in IRMOF-8 at T = 110K and P = 1385 Pa (3×3×1 unit cells). The snapshots illustrate the spatial distribution of CO_2 molecules within the framework. Adsorption is clearly non-uniform, with localized regions of higher density and clustering observed from both perspectives. This

inhomogeneous distribution may result from cooperative adsorption behavior and preferential site occupation within the pore network. (B) CH_4 adsorption in IRMOF-8 at 230 K, P = 141 kPa (3×3×1 unit cells). A less pronounced inhomogeneous distribution is observed, due to the pores being only partially filled and the accessible volume being reduced, limiting the ability of the gas to fluctuate within the equilibrium region, as shown in Fig. 6 (D).

Experimental setup

Comment on measurement accuracy

High precision in the adsorption measurements was ensured by carefully optimizing the experimental conditions. A sample mass of 32.2 mg was used, with a calibrated dead volume of 41.44 cm^3 and a total surface area per adsorption cell was 121.2 m^2 (more than twice the recommended value). As a result, the recorded isotherms are smooth, free from outliers, indicating both excellent temperature stability and sufficient sample loading to accurately capture adsorption behavior.

Experimental isotherms (AIF format)

CO_2

----------- 195 K -----------------------------------
data_BELMaster_v7423
_exptl_operator 'Volodymyr'
_exptl_date 2021-07-26T00:00:00
_exptl_instrument 'BEL 00218'
_exptl_adsorptive 'CO2'
_exptl_temperature 195.0
_adsnt_sample_mass 0.0322
_adsnt_sample_id 'glovebox'
_adsnt_material_id 'MOF-5-CO2-195K'
_units_temperature 'K'
_units_pressure 'kPa'
_units_mass 'g'
_units_loading 'ml(STP) g-1'

loop_
_adsorp_pressure
_adsorp_p0
_adsorp_amount
0.044805 101.0 0.2717
0.092143 101.0 0.5557
0.1409 101.0 0.8426

0.191 101.0 1.1371
0.2418 101.0 1.431
0.2973 101.0 1.7501
0.3547 101.0 2.0779
0.4097 101.0 2.3895
0.4683 101.0 2.7228
0.5207 101.0 3.0206
0.587 101.0 3.3915
0.6479 101.0 3.7353
0.7116 101.0 4.0934
0.7802 101.0 4.4755
0.8336 101.0 4.7768
0.9032 101.0 5.1655
0.9608 101.0 5.4855
1.0905 101.0 6.1713
1.2074 101.0 6.8547
1.3218 101.0 7.5923
1.4521 101.0 8.3601
1.5987 101.0 9.0955
1.7005 101.0 9.7035
1.8431 101.0 10.506
1.9612 101.0 11.135
2.0711 101.0 11.737
2.234 101.0 12.641
2.3684 101.0 13.447
2.5028 101.0 14.126
2.625 101.0 14.86
2.7594 101.0 15.564
2.8978 101.0 16.325
3.0363 101.0 17.127
3.2073 101.0 18.085
3.3499 101.0 18.904
3.5291 101.0 19.923
3.692 101.0 20.78
3.8874 101.0 21.929
4.0707 101.0 22.877
4.2825 101.0 24.107
4.482 101.0 25.189
4.7264 101.0 26.482
4.9504 101.0 27.743
5.211 101.0 29.186
5.5857 101.0 31.389
5.7933 101.0 32.45
6.0621 101.0 33.978

6.4857 101.0 36.376
6.7137 101.0 37.712
7.1576 101.0 40.199
7.4345 101.0 41.822
7.9192 101.0 44.635
8.3427 101.0 47.207
8.7703 101.0 49.77
9.2183 101.0 52.466
9.6907 101.0 55.288
10.42 101.0 59.827
11.039 101.0 63.704
11.617 101.0 67.472
12.199 101.0 71.316
12.794 101.0 75.332
13.401 101.0 79.636
14.04 101.0 84.346
14.7 101.0 89.388
15.392 101.0 94.966
16.101 101.0 101.13
17.245 101.0 112.31
17.807 101.0 118.62
18.784 101.0 132.23
18.947 101.0 154.19
18.964 101.0 175.86
18.972 101.0 197.36
18.976 101.0 218.72
18.976 101.0 239.95
18.984 101.0 271.15
18.988 101.0 302.04
18.996 101.0 322.62
18.996 101.0 343.15
18.992 101.0 363.43
18.996 101.0 392.65
19.004 101.0 421.47
19.004 101.0 449.28
19.013 101.0 476.55
19.017 101.0 503.05
19.029 101.0 528.42
19.045 101.0 553.07
19.106 101.0 576.26
19.232 101.0 603.07
19.509 101.0 627.28
19.896 101.0 642.78
21.012 101.0 653.47

21.855 101.0 659.38
22.938 101.0 665.75
24.168 101.0 671.92
25.52 101.0 677.77
26.933 101.0 683.07
28.383 101.0 687.91
29.939 101.0 692.54
31.499 101.0 696.75
33.156 101.0 700.69
34.903 101.0 704.56
36.748 101.0 708.3
38.67 101.0 711.81
40.702 101.0 715.34
42.824 101.0 718.58
45.068 101.0 721.76
47.422 101.0 724.82
49.922 101.0 727.87
52.521 101.0 730.76
55.253 101.0 733.5
58.124 101.0 736.12
61.171 101.0 739.16
64.343 101.0 741.72
67.727 101.0 744.3
71.242 101.0 746.47
74.976 101.0 748.9
78.89 101.0 750.85
82.991 101.0 753.17
87.324 101.0 755.43
91.901 101.0 757.38
96.572 101.0 759.56

loop_
_desorp_pressure
_desorp_p0
_desorp_amount
95.078 101.0 758.89
90.411 101.0 757.03
85.907 101.0 754.86
81.578 101.0 752.77
77.509 101.0 750.28
73.604 101.0 748.11
69.91 101.0 745.7
66.408 101.0 743.2
63.072 101.0 740.72

59.896	101.0	738.03
56.899	101.0	735.24
54.04	101.0	732.44
51.336	101.0	729.53
48.746	101.0	726.59
46.306	101.0	723.4
43.985	101.0	720.3
41.778	101.0	717.01
39.692	101.0	713.55
37.693	101.0	710.11
35.815	101.0	706.46
34.024	101.0	702.71
32.334	101.0	698.77
30.721	101.0	694.78
29.202	101.0	690.45
27.768	101.0	685.89
26.404	101.0	681.21
25.113	101.0	676.22
23.899	101.0	670.85
22.763	101.0	665.01
21.713	101.0	658.71
19.102	101.0	635.74
18.956	101.0	611.02
18.947	101.0	586.42
18.947	101.0	561.97
18.947	101.0	537.75
18.943	101.0	513.72
18.939	101.0	489.86
18.927	101.0	466.2
18.931	101.0	442.47
18.927	101.0	418.98
18.919	101.0	395.73
18.915	101.0	372.76
18.903	101.0	340.9
18.894	101.0	309.58
18.886	101.0	279.09
18.882	101.0	249.49
18.846	101.0	220.43
18.813	101.0	192.22
18.732	101.0	164.93
18.524	101.0	139.58
17.436	101.0	114.87
15.266	101.0	94.183
14.793	101.0	90.256

14.174 101.0 85.414
13.486 101.0 80.302
12.79 101.0 75.297
12.093 101.0 70.614
11.43 101.0 66.248
10.802 101.0 62.155
10.212 101.0 58.357
9.6337 101.0 54.908
9.5074 101.0 54.041
9.0513 101.0 51.267
8.5952 101.0 48.553
8.1594 101.0 45.944
7.74 101.0 43.504
7.3409 101.0 41.169
6.9662 101.0 38.876
6.2902 101.0 35.079
5.663 101.0 31.427
5.439 101.0 30.296
5.0888 101.0 28.265
4.8974 101.0 27.202
4.0096 101.0 22.214
3.5983 101.0 20.008
3.411 101.0 18.982
3.2318 101.0 18.009
3.0607 101.0 17.111
2.9019 101.0 16.204
2.7512 101.0 15.311
2.6005 101.0 14.518
2.4621 101.0 13.717
2.3277 101.0 13.012
2.2055 101.0 12.299
2.0793 101.0 11.669
1.9775 101.0 10.981
1.8634 101.0 10.382
1.7087 101.0 9.6124
1.5906 101.0 8.9832
1.448 101.0 8.2894
1.3422 101.0 7.6953
1.2254 101.0 6.9599
1.101 101.0 6.2502
0.982 101.0 5.5703
0.917 101.0 5.1974
0.8611 101.0 4.877
0.7897 101.0 4.4684

0.7344 101.0 4.1468
0.6776 101.0 3.8182
0.6317 101.0 3.5545
0.5763 101.0 3.2362
0.5336 101.0 2.986
0.4849 101.0 2.7017
0.4358 101.0 2.4137
0.3886 101.0 2.1351
0.3442 101.0 1.8746
0.2957 101.0 1.5895
0.2461 101.0 1.2949
0.202 101.0 1.0302
0.1632 101.0 0.7938
0.1322 101.0 0.6012
0.1081 101.0 0.4506
0.088823 101.0 0.3291

-------------- 200 K ---------------------------
data_BELMaster_v7423
_exptl_operator 'Volodymyr'
_exptl_date 2021-07-26T00:00:00
_exptl_instrument 'BEL 00218'
_exptl_adsorptive 'CO2'
_exptl_temperature 200.0
_adsnt_sample_mass 0.0322
_adsnt_sample_id 'glovebox'
_adsnt_material_id 'MOF-5-CO2-200K'
_units_temperature 'K'
_units_pressure 'kPa'
_units_mass 'g'
_units_loading 'ml(STP) g-1'

loop_
_adsorp_pressure
_adsorp_amount
1.024 4.488
5.65 23.447
10.137 42.159
14.45 61.414
18.436 81.554
22.32 105.814
25.356 133.106
26.383 178.885
26.435 230.695

26.475 285.72
26.511 335.069
26.544 386.551
26.59 438.49
26.668 490.747
26.766 540.96
27.43 592.556
30.759 618.674
35.273 637.214
39.761 649.853
45.019 660.95
49.953 669.119
54.724 675.916
60.017 682.107
65.172 687.577
69.968 691.943
75.43 696.331
80.928 700.342
86.213 703.805
91.463 707.069
95.529 711.886
99.95 716.208
103.374 717.997
128.732 728.176
154.133 736.798

loop_
_desorp_pressure
_desorp_amount
125.602 728.242
97.936 716.834
91.361 710.474
84.876 706.894
78.315 702.712
72.17 698.256
66.02 693.124
60.078 687.375
54.357 680.943
48.842 673.328
43.518 664.346
38.511 653.492
33.909 640.051
29.836 622.464
26.657 598.14

26.092 553.292
26.083 504.022
26.068 454.815
26.047 405.603
26.022 356.403
25.997 307.349
25.963 258.276
25.911 209.288
25.703 161.619
23.87 126.779
20.69 102.701
17.107 81.977
10.481 50.727
6.262 32.895
3.715 22.467
2.207 16.28
1.319 12.553
0.797 10.298

-------------- 210 K ------------------------------------
data_BELMaster_v7423
_exptl_operator 'Volodymyr'
_exptl_date 2021-07-26T00:00:00
_exptl_instrument 'BEL 00218'
_exptl_adsorptive 'CO2'
_exptl_temperature 210.0
_adsnt_sample_mass 0.0322
_adsnt_sample_id 'glovebox'
_adsnt_material_id 'MOF-5-CO2-210K'
_units_temperature 'K'
_units_pressure 'kPa'
_units_mass 'g'
_units_loading 'ml(STP) g-1'

loop_
_adsorp_pressure
_adsorp_amount
0.637 1.769
5.054 12.736
9.57 23.887
14.208 35.547
18.649 47.153
22.745 58.484
26.813 70.537

30.956 83.973
34.467 96.918
38.422 114.101
41.513 130.781
44.077 148.896
46.3 172.558
47.606 201.006
47.827 244.197
47.878 282.036
47.928 320.461
47.967 354.75
48.02 393.491
48.127 434.873
48.287 475.764
48.643 511.964
49.997 545.5
52.498 567.001
55.386 582.406
58.958 595.722
62.947 606.877
66.794 615.449
70.47 622.338
74.229 628.479
78.382 634.395
82.253 639.226
86.022 643.578
89.825 647.619
93.616 651.226
98.245 648.95
102.242 652.08
127.346 668.345
151.387 678.321
176.006 685.809
199.69 695.48
223.44 699.775
247.926 704.278
265.067 709.334
294.093 715.259
322.682 720.29

loop_
_desorp_pressure
_desorp_amount
290.904 717.146

258.675 711.452
227.38 708.437
196.781 700.393
167.762 692.372
138.473 682.617
110.938 667.946
85.011 644.951
78.045 642.202
72.31 634.282
66.822 625.058
61.494 613.629
56.755 599.802
52.514 581.49
49.203 556.494
47.245 521.062
47.163 471.107
47.126 420.632
47.061 370.072
46.973 319.848

CH_4

------------ 92 K ---
data_BELMaster_v7423
_exptl_operator 'Volodymyr'
_exptl_date 2021-07-29T00:00:00
_exptl_instrument 'BEL 00218'
_exptl_adsorptive 'CH4'
_exptl_temperature 92.0
_adsnt_sample_mass 0.0322
_adsnt_sample_id 'glovebox'
_adsnt_material_id 'MOF-5-CH4-92K'
_units_temperature 'K'
_units_pressure 'kPa'
_units_mass 'g'
_units_loading 'ml(STP) g-1'

loop_
_adsorp_pressure
_adsorp_p0
_adsorp_amount
4.8511E-05 13.8 0.0020351

9.0974E-05 13.8 0.2873
0.00012259 13.8 0.53158
0.00015089 13.8 0.77562
0.00017848 13.8 1.0254
0.00020509 13.8 1.2757
0.00023054 13.8 1.5255
0.00025582 13.8 1.7757
0.00028088 13.8 2.0258
0.00030696 13.8 2.2862
0.00033611 13.8 2.5702
0.00035987 13.8 2.818
0.00038494 13.8 3.073
0.00040942 13.8 3.3255
0.00043356 13.8 3.5736
0.00045845 13.8 3.8265
0.00048278 13.8 4.0769
0.00051174 13.8 4.3579
0.00053651 13.8 4.6105
0.00056148 13.8 4.8655
0.00063661 13.8 5.635
0.0008414 13.8 7.6854
0.0010489 13.8 9.6925
0.0012613 13.8 11.703
0.0014951 13.8 13.852
0.0017306 13.8 15.971
0.0019629 13.8 18.061
0.002205 13.8 20.156
0.002456 13.8 22.246
0.0027427 13.8 24.287
0.0030025 13.8 26.405
0.0032598 13.8 28.642
0.0035353 13.8 30.749
0.0038315 13.8 32.877
0.0041247 13.8 34.969
0.0044377 13.8 37.093
0.004764 13.8 39.228
0.0051044 13.8 41.39
0.0054505 13.8 43.496
0.0058361 13.8 45.758
0.0062249 13.8 47.933
0.006631 13.8 50.115
0.0070496 13.8 52.264
0.0075017 13.8 54.46
0.0079325 13.8 56.492

0.0084207 13.8 58.67
0.0089557 13.8 60.94
0.0095047 13.8 63.12
0.010081 13.8 65.335
0.010813 13.8 68.302
0.014696 13.8 80.006
0.019666 13.8 91.701
0.025859 13.8 103.41
0.032696 13.8 114.97
0.039311 13.8 126.55
0.045089 13.8 138.15
0.047197 13.8 149.85
0.047197 13.8 161.79
0.047156 13.8 173.71
0.047197 13.8 185.58
0.047197 13.8 197.54
0.04728 13.8 209.43
0.047238 13.8 221.37
0.04728 13.8 233.3
0.047238 13.8 245.19
0.047321 13.8 257.08
0.04728 13.8 268.99
0.047321 13.8 280.87
0.047362 13.8 292.77
0.04728 13.8 304.71
0.047321 13.8 316.58
0.047321 13.8 328.44
0.047362 13.8 340.33
0.047362 13.8 352.29
0.047362 13.8 364.12
0.047404 13.8 375.94
0.047321 13.8 387.84
0.047362 13.8 399.66
0.047445 13.8 411.56
0.047362 13.8 423.43
0.047404 13.8 435.38
0.047487 13.8 447.24
0.047487 13.8 459.11
0.047569 13.8 470.94
0.047445 13.8 482.86
0.047445 13.8 494.74
0.047487 13.8 506.59
0.047487 13.8 518.49
0.047487 13.8 530.34

0.047487 13.8 542.15
0.047528 13.8 553.97
0.047528 13.8 565.81
0.047611 13.8 577.7
0.047569 13.8 589.5
0.047652 13.8 601.29
0.047735 13.8 613.11
0.0479 13.8 624.93
0.047983 13.8 636.84
0.048272 13.8 648.62
0.049223 13.8 660.52
0.05183 13.8 672.28
0.053402 13.8 684.15
0.061389 13.8 695.86
0.074536 13.8 707.44
0.095896 13.8 719.11
0.12047 13.8 727.79
0.14296 13.8 733.54
0.20783 13.8 744.64
0.25737 13.8 750.32
0.31968 13.8 755.78
0.39921 13.8 761.06
0.49941 13.8 766.18
0.6242 13.8 771.17
0.7778 13.8 775.91
0.96613 13.8 780.53
1.172 13.8 784.58
1.4252 13.8 788.69
1.7102 13.8 792.2
2.0074 13.8 795.43
2.3657 13.8 798.34
2.7688 13.8 801.21
3.237 13.8 804.24
3.6441 13.8 806.3
4.0838 13.8 808.25
4.5276 13.8 810.01
5.0365 13.8 811.89
5.6024 13.8 813.59
6.2456 13.8 815.46
6.9499 13.8 817.18
7.7275 13.8 819.08
8.2445 13.8 820.09
9.1321 13.8 821.77
9.7183 13.8 822.71

10.264 13.8 823.57
10.846 13.8 824.55
11.461 13.8 825.38
12.104 13.8 826.59
12.764 13.8 827.89
13.415 13.8 830.22

loop_
_desorp_pressure
_desorp_p0
_desorp_amount
12.926 13.8 828.84
11.909 13.8 826.52
11.155 13.8 825.24
10.484 13.8 824.11
9.8771 13.8 823.01
9.2746 13.8 822.2
8.7453 13.8 821.24
8.2201 13.8 820.23
7.357 13.8 818.4
6.8685 13.8 817.45
6.1397 13.8 815.55
5.4354 13.8 813.81
4.8207 13.8 811.72
4.2629 13.8 809.69
3.7622 13.8 807.6
3.3184 13.8 805.51
2.9357 13.8 803.26
2.5815 13.8 801.01
2.1743 13.8 798.23
1.816 13.8 795.07
1.5147 13.8 791.56
1.2649 13.8 787.79
1.0727 13.8 784.44
0.92727 13.8 781.43
0.81439 13.8 778.71
0.72423 13.8 776.22
0.65196 13.8 773.96
0.59185 13.8 771.86
0.54215 13.8 769.92
0.46392 13.8 766.44
0.43293 13.8 764.86
0.19336 13.8 744.72
0.10478 13.8 724.66

0.082132 13.8 714.42
0.067097 13.8 704.35
0.057458 13.8 694.27
0.050837 13.8 684.16
0.047073 13.8 674.11
0.047197 13.8 663.97
0.047073 13.8 653.84
0.047073 13.8 643.7
0.047114 13.8 633.57
0.047114 13.8 623.44
0.047032 13.8 613.28
0.047197 13.8 603.15
0.047158 13.8 593.02
0.047073 13.8 582.86
0.047156 13.8 572.73
0.047156 13.8 562.6
0.047114 13.8 552.56
0.047156 13.8 542.41
0.047197 13.8 532.24
0.047238 13.8 492.14
0.047321 13.8 451.99
0.047197 13.8 411.82
0.047445 13.8 371.74
0.061926 13.8 331.35
0.046864 13.8 331.33

------------- 102 K -----------------------------------
data_BELMaster_v7423
_exptl_operator 'Volodymyr'
_exptl_date 2021-07-27T00:00:00
_exptl_instrument 'BEL 00218'
_exptl_adsorptive 'CH4'
_exptl_temperature 102.0
_adsnt_sample_mass 0.0322
_adsnt_sample_id 'glovebox'
_adsnt_material_id 'MOF-5-CH4-102K'
_units_temperature 'K'
_units_pressure 'kPa'
_units_mass 'g'
_units_loading 'ml(STP) g-1'

loop_
_adsorp_pressure
_adsorp_p0

_adsorp_amount
7E-05 42.16 0.00050234
0.00022529 42.16 0.27564
0.00037788 42.16 0.52272
0.0005331 42.16 0.77358
0.00068244 42.16 1.0155
0.0010082 42.16 1.5455
0.0020691 42.16 3.2698
0.0034344 42.16 5.4523
0.0047474 42.16 7.5058
0.0061199 42.16 9.5979
0.0076003 42.16 11.783
0.0091505 42.16 13.998
0.010517 42.16 16
0.012178 42.16 18.216
0.013643 42.16 20.237
0.015283 42.16 22.256
0.017023 42.16 24.325
0.018782 42.16 26.399
0.020677 42.16 28.5
0.022549 42.16 30.62
0.024553 42.16 32.651
0.026732 42.16 34.86
0.028921 42.16 36.992
0.03112 42.16 39.145
0.033409 42.16 41.229
0.035785 42.16 43.292
0.038167 42.16 45.361
0.041789 42.16 48.333
0.057514 42.16 59.856
0.076051 42.16 71.356
0.09779 42.16 82.782
0.12302 42.16 94.441
0.14337 42.16 103.19
0.16428 42.16 112.03
0.1846 42.16 120.86
0.20404 42.16 129.73
0.22717 42.16 141.68
0.24694 42.16 153.9
0.26267 42.16 166.09
0.27475 42.16 178.34
0.28385 42.16 190.83
0.2867 42.16 203.82
0.28674 42.16 216.76

0.28666 42.16 229.79
0.28674 42.16 242.77
0.28674 42.16 255.79
0.28674 42.16 268.72
0.28686 42.16 281.7
0.28686 42.16 294.68
0.28698 42.16 307.65
0.28698 42.16 320.56
0.28706 42.16 333.49
0.28714 42.16 346.47
0.28714 42.16 359.47
0.28722 42.16 372.49
0.28718 42.16 385.45
0.28727 42.16 398.36
0.28731 42.16 411.34
0.28735 42.16 424.37
0.28735 42.16 437.29
0.28747 42.16 450.22
0.28763 42.16 463.22
0.28763 42.16 476.07
0.28763 42.16 489
0.28767 42.16 501.94
0.28783 42.16 514.9
0.288 42.16 527.73
0.28836 42.16 540.64
0.28905 42.16 553.39
0.28987 42.16 566.2
0.2908 42.16 578.94
0.29295 42.16 591.65
0.29624 42.16 604.16
0.30112 42.16 616.5
0.30949 42.16 629.33
0.3252 42.16 641.73
0.34802 42.16 653.98
0.37116 42.16 663.07
0.3904 42.16 669.1
0.41401 42.16 675.15
0.4753 42.16 686.63
0.566 42.16 698.02
0.62895 42.16 703.84
0.7019 42.16 709.25
0.79008 42.16 714.56
0.89668 42.16 719.85
1.0324 42.16 725.26

1.1881 42.16 730.22
1.3682 42.16 735.43
1.584 42.16 739.99
1.8486 42.16 744.4
2.1255 42.16 748.13
2.4675 42.16 752.05
2.8054 42.16 755.19
3.1515 42.16 758.19
3.5138 42.16 760.68
3.9087 42.16 763.45
4.3484 42.16 765.77
4.8451 42.16 768.35
5.3825 42.16 770.65
5.9973 42.16 773.28
6.6853 42.16 775.58
7.4629 42.16 777.91
7.9718 42.16 779.42
8.8227 42.16 781.53
9.3804 42.16 783.1
9.9382 42.16 784.07
10.484 42.16 785.4
11.074 42.16 786.53
11.721 42.16 787.66
12.369 42.16 788.83
13.069 42.16 790.06
13.814 42.16 791.37
14.62 42.16 792.52
15.459 42.16 793.8
16.358 42.16 794.77
17.278 42.16 796.04
18.292 42.16 797.05
19.33 42.16 798.4
20.45 42.16 799.49
21.626 42.16 800.86
22.888 42.16 802.06
24.208 42.16 803.36
25.669 42.16 804.44
27.11 42.16 805.7
28.735 42.16 806.95
30.363 42.16 808.19
32.134 42.16 809.46
33.995 42.16 810.84
35.977 42.16 812.36
38.078 42.16 813.95

40.252 42.16 816.16
41.31 42.16 818.31

loop_
_desorp_pressure
_desorp_p0
_desorp_amount
40.398 42.16 817.15
39.307 42.16 815.6
37.113 42.16 813.61
34.976 42.16 811.89
32.981 42.16 810.48
31.108 42.16 809.1
29.337 42.16 807.92
27.668 42.16 806.72
26.097 42.16 805.33
24.611 42.16 804.1
23.206 42.16 802.91
21.879 42.16 801.68
20.633 42.16 800.48
19.452 42.16 799.33
18.341 42.16 798.08
17.278 42.16 796.92
16.301 42.16 795.47
15.365 42.16 794.33
14.465 42.16 793.16
13.639 42.16 791.84
12.849 42.16 790.7
12.104 42.16 789.48
11.375 42.16 788.22
10.736 42.16 786.94
10.109 42.16 785.77
9.5107 42.16 784.54
8.9733 42.16 783.06
8.4237 42.16 781.77
7.9352 42.16 780.5
7.1209 42.16 778.11
6.6283 42.16 776.66
5.9566 42.16 774.15
5.2767 42.16 771.52
4.6823 42.16 768.76
4.1489 42.16 765.96
3.6685 42.16 763.21
3.2411 42.16 760.22

2.8746 42.16 757.17
2.5408 42.16 754.07
2.2314 42.16 751.02
1.9219 42.16 746.88
1.6858 42.16 743.04
1.4781 42.16 739.25
1.2908 42.16 734.75
1.1422 42.16 730.72
1.0281 42.16 726.98
0.93716 42.16 723.51
0.80211 42.16 717.19
0.75094 42.16 714.29
0.7075 42.16 711.55
0.49525 42.16 691.24

---------- 110 K ------------------------------
data_BELMaster_v7423
_exptl_operator 'Volodymyr'
_exptl_date 2021-07-26T00:00:00
_exptl_instrument 'BEL 00218'
_exptl_adsorptive 'CH4'
_exptl_temperature 110.0
_adsnt_sample_mass 0.0322
_adsnt_sample_id 'glovebox'
_adsnt_material_id 'MOF-5-CH4-110K'
_units_temperature 'K'
_units_pressure 'kPa'
_units_mass 'g'
_units_loading 'ml(STP) g-1'

loop_
_adsorp_pressure
_adsorp_p0
_adsorp_amount
6.9556E-05 81.14 0.00055939
0.00050244 81.14 0.24861
0.0010036 81.14 0.49775
0.0019816 81.14 0.97087
0.0049253 81.14 2.3588
0.0080277 81.14 3.7761
0.011731 81.14 5.4446
0.015679 81.14 7.1119
0.019822 81.14 8.8068
0.024369 81.14 10.599

0.029139 81.14 12.434
0.034606 81.14 14.491
0.038707 81.14 15.966
0.043478 81.14 17.717
0.049329 81.14 19.762
0.054485 81.14 21.555
0.060426 81.14 23.541
0.065499 81.14 25.197
0.071477 81.14 27.19
0.077327 81.14 29.066
0.087821 81.14 32.285
0.10421 81.14 37.064
0.12249 81.14 42.167
0.14249 81.14 47.448
0.16418 81.14 52.833
0.18689 81.14 58.154
0.20327 81.14 61.78
0.22919 81.14 67.265
0.25616 81.14 72.713
0.28769 81.14 78.658
0.32067 81.14 84.67
0.33659 81.14 87.661
0.35108 81.14 90.705
0.38282 81.14 96.707
0.41844 81.14 102.88
0.45344 81.14 108.85
0.47088 81.14 111.85
0.50595 81.14 117.88
0.52314 81.14 120.93
0.55752 81.14 127.15
0.58999 81.14 133.3
0.62108 81.14 139.27
0.66461 81.14 148.33
0.6914 81.14 154.54
0.73828 81.14 167.08
0.77673 81.14 179.71
0.80704 81.14 192.95
0.83026 81.14 205.45
0.86417 81.14 231.39
0.88131 81.14 251.85
0.89253 81.14 271.93
0.90185 81.14 298.24
0.90712 81.14 324.23
0.91068 81.14 350.2

0.91287 81.14 371.28
0.91498 81.14 392.42
0.91725 81.14 413.46
0.92037 81.14 434.41
0.92425 81.14 455.07
0.92956 81.14 475.48
0.93706 81.14 495.72
0.95249 81.14 522.21
0.97668 81.14 547.82
1.0051 81.14 567.87
1.043 81.14 586.85
1.1202 81.14 612.22
1.1764 81.14 624.89
1.2298 81.14 637.74
1.3316 81.14 650.07
1.3926 81.14 656.17
1.47 81.14 662.38
1.6288 81.14 673.76
1.7469 81.14 679.88
1.869 81.14 685.81
2.0156 81.14 691.28
2.1703 81.14 696.34
2.3739 81.14 701.92
2.5856 81.14 706.63
2.8462 81.14 711.81
3.1067 81.14 716.07
3.4283 81.14 720.44
3.8233 81.14 725.09
4.1327 81.14 728.5
4.5805 81.14 732.33
5.0935 81.14 736.16
5.4965 81.14 738.82
6.0502 81.14 742.17
6.5021 81.14 744.39
7.1616 81.14 747.57
7.6624 81.14 749.8
8.4766 81.14 752.57
9.0547 81.14 754.58
9.5636 81.14 756.36
10.125 81.14 758.06
10.712 81.14 759.41
11.314 81.14 760.96
11.941 81.14 762.56
12.617 81.14 764.27

13.342 81.14 765.72
14.107 81.14 767.17
14.921 81.14 768.55
15.78 81.14 770.04
16.684 81.14 771.59
17.649 81.14 773.09
18.659 81.14 774.47
19.741 81.14 775.88
20.873 81.14 777.3
22.086 81.14 778.74
23.361 81.14 780.28
24.729 81.14 781.59
26.166 81.14 782.88
27.737 81.14 784.32
29.329 81.14 785.93
31.002 81.14 787.32
32.814 81.14 788.95
34.727 81.14 790.39
36.759 81.14 791.83
38.904 81.14 793.4
41.192 81.14 794.9
43.59 81.14 796.56
46.139 81.14 798.28
48.85 81.14 800.09
51.712 81.14 801.49
54.753 81.14 803.18
57.941 81.14 805.16
61.357 81.14 807.04
64.952 81.14 809.09
68.782 81.14 811.11
72.813 81.14 813.36
77.1 81.14 815.35
79.196 81.14 816.52

loop_
_desorp_pressure
_desorp_p0
_desorp_amount
78.158 81.14 816.04
76.005 81.14 815.07
71.811 81.14 812.83
67.769 81.14 810.3
63.942 81.14 808.49
60.331 81.14 806.52

56.935 81.14 804.45
53.715 81.14 802.65
50.686 81.14 800.78
47.816 81.14 798.93
45.121 81.14 797.15
42.564 81.14 795.6
40.158 81.14 794.09
37.883 81.14 792.57
35.737 81.14 791.22
33.714 81.14 789.49
31.8 81.14 788.22
29.997 81.14 786.61
28.287 81.14 785.05
26.679 81.14 783.6
25.164 81.14 782.16
23.727 81.14 780.78
22.384 81.14 779.19
21.097 81.14 777.78
19.896 81.14 776.27
18.76 81.14 774.74
17.686 81.14 773.29
16.672 81.14 771.84

1. Li, H.; Eddaoudi, M.; O'Keeffe, M.; Yaghi, O. M. Design and Synthesis of an Exceptionally Stable and Highly Porous Metal–Organic Framework. Nature 1999, 402, 276–279.
2. Dubbeldam, D.; Calero, S.; Ellis, D. E.; Snurr, R. Q. RASPA: Molecular Simulation Software for Adsorption and Diffusion in Flexible Nanoporous Materials. Mol. Simul. 2016, 42, 81–101.
3. Mayo, S. L.; Olafson, B. D.; Goddard, W. A. DREIDING: A Generic Force Field for Molecular Simulations. J. Phys. Chem. 1990, 94, 8897–8909.
4. Rappe, A. K.; Casewit, C. J.; Colwell, K. S.; Goddard, W. A.; Skiff, W. M. UFF, a Full Periodic Table Force Field for Molecular Mechanics and Molecular Dynamics Simulations. J. Am. Chem. Soc. 1992, 114, 10024–10035.
5. Martin, M. G.; Siepmann, J. I. Transferable Potentials for Phase Equilibria. 1. United-Atom Description of n-Alkanes. J. Phys. Chem. B 1998, 102, 2569–2577.
6. Kancharlapalli, S.; et al. Fast and Accurate Machine Learning Strategy for Calculating Partial Atomic Charges in Metal–Organic Frameworks. J. Chem. Theory Comput. 2021, 17, 3052–3064.
7. Walton, K. S.; Millward, A. C.; Dubbeldam, D.; Frost, H.; Low, J. J.; Yaghi, O. M.; Snurr, R. Q. Understanding Inflections and Steps in Carbon Dioxide Adsorption Isotherms in Metal–Organic Frameworks. J. Am. Chem. Soc. 2008, 130, 406–407.
8. Dubbeldam, D.; Walton, K. S.; Ellis, D. E.; Snurr, R. Q. Exceptional Negative Thermal Expansion in Isoreticular Metal–Organic Frameworks. Angew. Chem., Int. Ed. 2007, 46, 4496–4499.
9. Datar, A.; Witman, M.; Lin, L.-C. Improving Computational Assessment of Porous Materials for Water Adsorption Applications via Flat Histogram Methods. J. Phys. Chem. C 2021, 125, 4253–4266.

10. Kuchta, B.; et al. Adsorption-Induced Structural Phase Transformation in Nanopores. Angew. Chem., Int. Ed. 2017, 56, 16461–16464.
